# Corals and Reef‐Dwelling Fish Regulate Carbon Storage and Cycling Processes in Coral Reef Ecosystems

**DOI:** 10.1002/advs.202520612

**Published:** 2026-04-17

**Authors:** Yiting Chen, Wenliang Zhou, Lan Qiu, Han Lai, Mingpan Huang, Qian Li, Wen Yu, Pei‐Yuan Qian, Fuwen Wei

**Affiliations:** ^1^ Southern Marine Science and Engineering Guangdong Laboratory (Guangzhou) Guangzhou China; ^2^ Department of Ocean Science and Otto Poon Center for Climate Resilience and Sustainability Hong Kong University of Science and Technology Hong Kong SAR China; ^3^ Laoshan Laboratory Guangdong Institute Guangzhou China; ^4^ Jiangxi Key Laboratory of Conservation Biology College of Forestry Jiangxi Agricultural University Nanchang China; ^5^ CAS Key Laboratory of Animal Ecology and Conservation Biology Institute of Zoology Chinese Academy of Sciences Beijing China

**Keywords:** biodiversity conservation, carbon cycling process, carbon reservoir, coral reef ecosystems, reef‐dwelling organisms, South China Sea

## Abstract

Coral reef ecosystems are among the most biodiverse and ecologically significant marine habitats; however, the carbon storage potential of these ecosystems and how corals and reef‐dwelling fish participate in carbon cycling remain largely unexplored. In this study, stereo‐video surveys, elemental analysis, and statistical modeling were used to quantify the carbon reservoirs of coral reef ecosystems in the South China Sea (SCS) in terms of carbon reservoirs in reef fish, coral communities, and sediment, and assess the factors influencing carbon storage. The results revealed that the average carbon stock of the SCS coral reef ecosystems is 3.22 kgC m^−2^, with an estimated carbon storage of 25.73–121.99 TgC. Sediments are the dominant carbon reservoir and store 90.6%–95.7% of the total estimated carbon stock; however, coral and fish biomass also contribute to carbon stocks. The impact of corals and reef‐dwelling fish on the carbon cycle of coral reefs far exceeds their direct carbon storage function, and reef fish communities transport an average of 235.63 gC m^−2^ yr^−1^ into the sediment carbon reservoir. These findings, reported for the first time, demonstrate the significant carbon sequestration potential of coral reef ecosystems, in which SCS reefs were used as an example, and provide critical insights into the role of corals and reef‐dwelling fish in coral reef carbon cycling. These findings further highlight the necessity of biodiversity conservation amid increasingly severe global changes, specifically to maintain the carbon sequestration function and stability of coral reef ecosystems.

## Graphical abstract

Coral reefs are biodiversity hotspots, yet their role in carbon storage and cycling remains poorly understood. Using field surveys and modeling in the South China Sea, we reveal the overlooked potential of carbon storage in reef ecosystems and how reef fish, corals, and surface sediment jointly shape reef carbon reservoirs. Our findings highlight the role of these organisms in regulating carbon dynamics and underscore the importance of biodiversity conservation for sustaining crucial ecosystem functions.

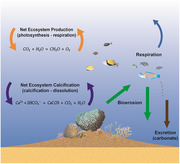

## 1 Introduction

Rising atmospheric carbon concentrations since the industrial revolution have intensified global warming, sea‐level rise, and ocean acidification, amplifying threats to ecosystems and human societies [1]. Climate policies for quantifying how ecosystems store, release, and redistribute carbon are urgently needed. Widespread research has advanced the understanding of blue carbon ecosystems (BCEs), such as mangroves, salt marshes, and seagrass meadows, which are now recognized as critical natural climate solutions. Mangroves can store ∼11.7 PgC globally, while seagrass meadows and tidal marshes collectively bury tens of teragrams of carbon annually [2, 3]. This recognition has placed BCEs at the forefront of global carbon mitigation strategies. In contrast to these traditional BCEs, coral reefs remain underrepresented in global carbon assessments, despite being among the most biodiverse ecosystems on Earth and supporting highly dynamic biogeochemical processes [4].

Debates have long centered on whether reefs function as net carbon sources [5, 6] or net carbon sinks [7, 8] or shift dynamically between these states depending on environmental conditions [9, 10]. Two core processes underpin these dynamics: net ecosystem production (NEP), which refers to the balance of photosynthesis and respiration, and net ecosystem calcification (NEC), which refers to the balance of calcification and dissolution [11, 12]. Calcification and respiration increase the seawater partial pressure of CO_2_ (*p*CO_2_) and dissolved inorganic carbon (DIC), which can decrease the pH and diminish the ocean's capacity to absorb atmospheric CO_2_. Conversely, the photosynthetic uptake of DIC and calcium carbonate (CaCO_3_) dissolution increases total alkalinity, which then decreases *p*CO_2_ and enhances seawater buffering capacity [13, 14]. Rather than revisiting the source‐versus‐sink debate, research could focus on assessing the magnitude of coral reef carbon reservoirs, how they are mediated by biological components, and the importance of their conservation. Reef‐building corals undeniably serve as long‐term inorganic carbon reservoirs because of the deposition of CaCO_3_ skeletons [15], and their symbiotic algae continuously fix carbon via photosynthesis [16]. Despite these insights, significant gaps remain concerning the carbon storage potential of coral reef ecosystems. Moreover, only a few studies have quantified the actual carbon stored within these systems, limiting the understanding of their role in marine carbon reservoirs.

First, coral reef ecosystems encompass a diverse array of organisms, including reef‐building corals, fish, and associated fauna, which collectively regulate carbon cycling via primary production, calcification, and sediment deposition [17]. These processes store carbon in organic and inorganic forms, and reef‐associated species mediate the balance between carbon fixation, storage, and release [18]. An important example is the coral reef parrotfish group, hailed as the “gardeners of coral reefs.” Not only does this group of fish maintain the health of the corals by scraping off the algae attached to the coral surfaces, but it also creates coral sand by eating unhealthy corals [19, 20]. Coral sands excreted by parrotfish eventually settle on the seabed and play a crucial role in regulating carbon sequestration by coral reef organisms. Second, fish and other reef‐dwelling species constantly perform various life activities, such as breathing, feeding, and excretion [18, 21], and the effects of these processes on the carbon storage of coral reef ecosystems cannot be ignored. For example, marine fish are a prominent source of ichthyocarbonates (high‐magnesium calcite crystals produced in marine teleost fish intestines) and contribute considerably to the inorganic carbon cycle via the excretion of such carbonates [22, 23, 24]. Third, interactions within reef food webs, such as predator–prey relationships among fish populations, increase carbon storage in reef sediments by promoting the accumulation of organic material [25]. Despite these contributions, researchers still fail to precisely define the contributions of animal communities, such as corals and reef‐dwelling fish, to ecosystem‐level carbon reservoirs. This knowledge gap systematically underestimates the role of coral and reef‐dwelling organisms in coral reef carbon cycling.

The South China Sea (SCS) provides a critical natural laboratory to address the aforementioned gap. The SCS region includes more than 200 coral islands and reefs, including coral communities in Nansha (NS), Xisha (XS), Zhongsha (ZS), Dongsha Islands, and coastal areas in Hainan (HN), Guangdong (GD), Taiwan, and Fujian [26]. Spanning ∼3.4 million km^2^, the SCS region supports 8,000–12 000 km^2^ of coral reefs and exceptional biodiversity, which includes >570 coral species and >2,800 reef‐associated fish species [27, 28]. Although the coral reef ecosystems in the region have been extensively investigated in terms of biodiversity, spatial distribution, and degradation caused by disturbances [29, 30, 31, 32], their capacity for carbon storage remains poorly characterized. Furthermore, although past studies have primarily examined CO_2_ fluxes across the air–sea interface [8], planktonic primary production [33], or integrative assessments of flux pathways [34], long‐term carbon reservoirs within reef biota and sediments are rarely quantified.

**FIGURE 1:**
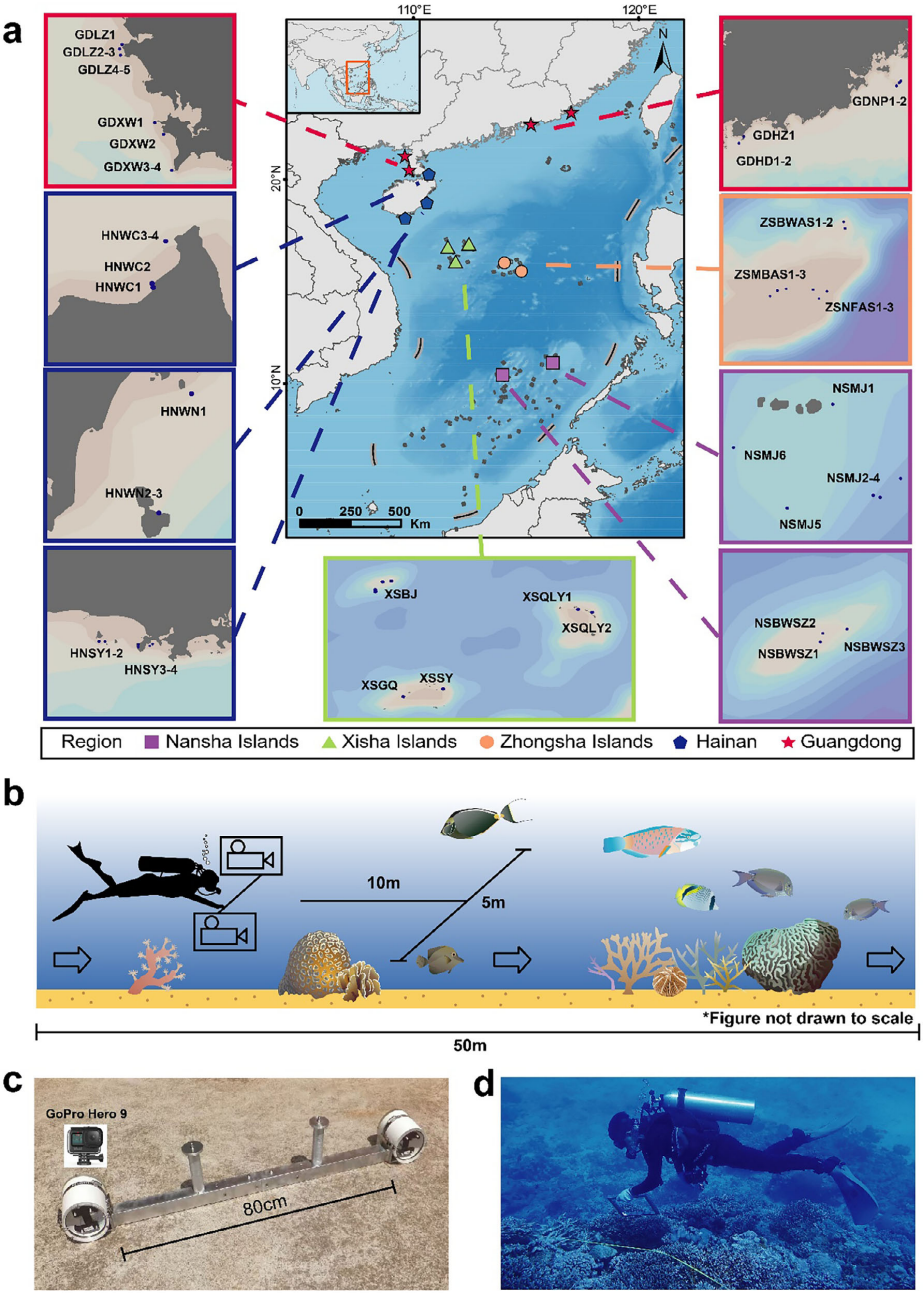
Geographic distribution of survey sites, methods, and equipment. (a) Map showing the survey locations in the South China Sea (Xisha, Zhongsha, and Nansha Islands; coastal Hainan and Guangdong). Location information is listed in Supplementary Table 2. NS = Nansha, ZS = Zhongsha, XS = Xisha, HN = Hainan, GD = Guangdong. (b) Stereo‐DOV (diver‐operated video) system method and settings of the belt transect. (c) Collapsible, swimmable DOV system from SeaGIS with GoPro cameras. (d) Photo of an underwater survey along a transect in Nansha.

Coral reef carbon cycling driven by climate change and anthropogenic pressures is experiencing accelerated alterations [35]; thus, the baseline magnitude and distribution of carbon reservoirs within these ecosystems must be robustly investigated. This study aims to provide the first comprehensive quantification of coral reef carbon storage in the SCS, with a focus on the roles of reef fish and coral communities in coral reef carbon cycling. A total of 17 reefs/atolls in the SCS region were surveyed, and species‐specific biomass, carbonate production, and sediment dynamics were integrated (Figure 1). The value of the carbon storage capacity of coral reefs in the SCS was assessed to not only inform regionally tailored conservation strategies but also ensure that these ecosystems can retain their role as biodiversity refuges in a rapidly changing climate.

## 2 Results

### 2.1 Fish Assemblage, Coral, and Sediment Data

**FIGURE 2:**
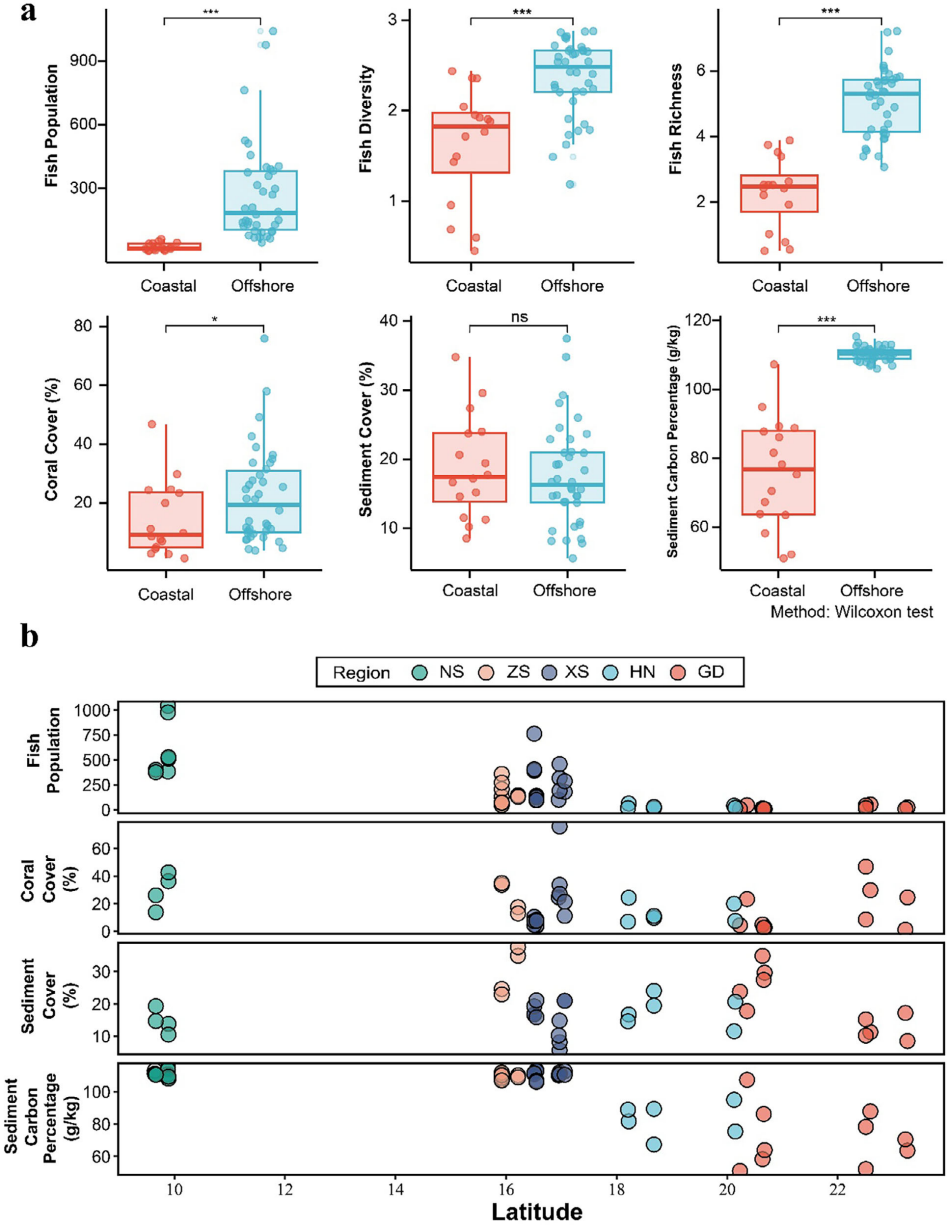
Transect survey results of the SCS coral reef ecosystem fish assemblage, coral, and sediment. (a) Comparison of survey results for fish population (total counts of fish), diversity (Shannon‐Wiener Index), richness (Margalef's Richness Index), coral cover, sediment cover, and sediment carbon percentage in coastal (HN and GD) and offshore (NS, ZS, and XS) regions. The bar represents the minimum to maximum values of each category. The horizontal line within the bar represents the median, and the whiskers represent the interquartile range. Statistically significant differences are denoted with * (^***^: *p* < 0.001, ^**^: *p* < 0.01, ^*^: *p* < 0.05, ns: nonsignificant). (b) Transect survey results of the fish population, coral cover, sediment cover, and sediment carbon percentage, with the x‐axis representing the latitude of each transect. Points are colored by region. NS = Nansha, ZS = Zhongsha, XS = Xisha, HN = Hainan, GD = Guangdong.

A total of 10 819 reef fish from 223 species were recorded on a camera at all survey locations. Ctenochaetus striatus, Thalassoma amblycephalum, Thalassoma quinqueevittatum, Acanthurus japonicus, Gnathodentex aureolineatus, Cephalopholis urodeta, Chromis vanderbilti, and Chromis margaritifer were the most common species. The fish family distribution is provided in Figure S1. Large numbers of Cirrhilabrus melanomarginatus and Caesio teres were also recorded, but they were found only in the Meiji Reef of NS and the Nanfei Ansha of ZS. Compared with the coastal regions (HN and GD), the offshore parts (NS, XS, and ZS) of the SCS had larger fish populations, fish diversity, fish richness, coral cover, and sediment carbon (SC) percentage (Figure 2a). In terms of abundance, the fish population in HN and GD accounted for only ∼3.6% of the total population surveyed at all locations. In terms of the fish population, the Meiji Reef in NS had the highest average (687.60), whereas that in Leizhou of GD had the lowest average (8.7). In terms of species richness, an average of 28 species was recorded on each transect video for NS, XS, and ZS, whereas only an average of 8 species was recorded for HN and GD. Similar patterns were detected for species richness, with the lowest in Leizhou (0.79 ± 0.23) and the highest in Meiji Reef (5.89 ± 1.30). In addition, the species recorded between the offshore and coastal regions did not clearly overlap. The biodiversity patterns of the surveyed locations were identical, with the highest in the Qilianyu Islands of XS (2.66 ± 0.31) and the lowest in Leizhou (0.70 ± 0.25), as determined by the Shannon–Wiener index. Comparisons of the fish indices of the five regions are shown in Figure S2. The average weight per fish was converted from the recorded fish length and grouped by genus. The average weight of the fish communities in most transects was ∼100 g of carbon per fish. The total carbon stock of each fish genus was calculated by multiplying the average weight by the biomass carbon fraction, and the results revealed that most of the fish genera had a carbon content of less than 25 g (Figures S3 and S4). The survey locations in the Shiyu Islands and Qilianyu Islands of XS had relatively high carbon contents per genus (30–75 g).

The OC and IC contents of 40 coral samples from 17 commonly found species in the SCS and 62 sediment samples were subsequently measured. The average carbon percentage of the coral was 116.80 ± 3.77 gC kg^−1,^ and that of the sediments was 100.15 ± 25.43 gC kg^−1^. In contrast to the fish assemblage data, the differences in SC percentages were not obvious across the transects in NS, XS, and ZS (Data S1), and they fluctuated mostly at ∼110 gC kg^−1^. These percentages in the coral reef ecosystems of offshore sites were not influenced by geographical location, animal community structure, or coral cover. However, given the massive deficiency in the coral and fish populations, the SC percentages of HN and GD were relatively low, averaging ∼76 gC kg^−1^. The average coral carbon percentage (116.80 gC kg^−1^) was used as P_C_ in Equation (3), whereas the average SC percentage (100.15 gC kg^−1^) was used as P_S_ in Equation (4). The details are provided in the Section 5.

### 2.2 Combined Ecosystem Carbon Stock of the SCS Coral Reef (in Total and by Region)

**FIGURE 3:**
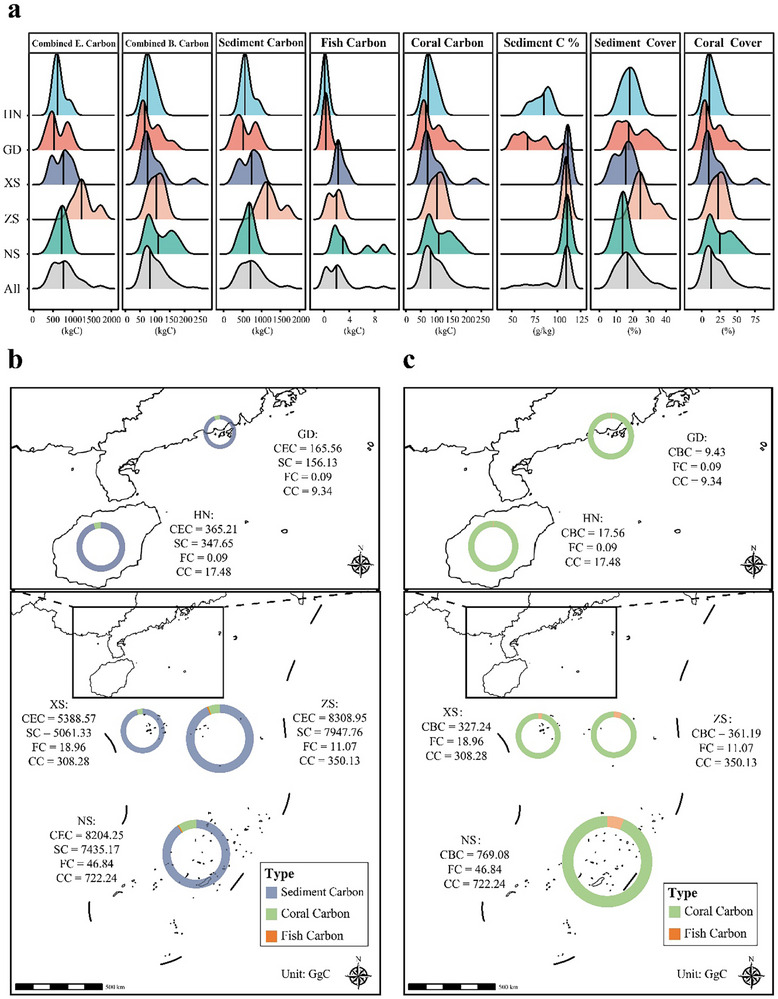
Estimation of South China Sea coral reef ecosystem carbon reservoirs by region and area. (a) Ridge plots of the estimated values of the transect‐level combined ecosystem carbon content, total biomass carbon content, sediment carbon content, fish assemblage carbon content, coral carbon content, sediment carbon percentage, and sediment and coral cover in each sampled area. The vertical lines within ridges indicate the median value for each category. (b, c) Pie charts of combined ecosystem carbon (sediment, coral, and fish) and combined biomass carbon (coral and fish) by region. CEC = combined ecosystem carbon, CBC = combined biomass carbon, SC = sediment carbon, FC = fish assemblage carbon, CC = coral carbon. Units are in gigagrams of carbon (GgC). Pie charts of XS, ZS, and NS are drawn to scale.

The combined ecosystem carbon (CEC) stock of the SCS region is 3.22 kgC m^−2^, where CEC is defined as the carbon stock in reef fish, corals, and surface sediment (Table S1). Multiplied by the total area of coral reefs in the region, the estimated CEC of the SCS coral reef ecosystems varied from 25 726.40 to 121 991.37 GgC. The highest CEC was recorded in ZS (8,308.95 GgC), followed by NS (8,204.25 GgC), XS (5,388.57 GgC), and HN (365.21 GgC), whereas the lowest CEC was recorded in GD (165.56 GgC) (Table S1). The coral reefs far from the mainland (NS, XS, and XS) composed most of the CEC in the SCS coral reef ecosystems (71%–85%) and the reef area (64%–80%). The disparities in SC stock across regions were due mainly to the variations in sediment area. Although NS has a larger reef area than ZS (2934.8 vs. 1628.2 km^2^, respectively), it had the smallest transect sediment cover (∼13.5%) and thus fell behind the transect CEC and SC measurements (Figure 3a). Similarly, although the regional CEC was greater in NS than in HN and GD (Figure 3b), the transect‐level CECs of the three regions were similar (all within the range of 600–700 kgC). Transect‐wise, the ZS region transects exhibited consistently higher levels of CEC, SC, and sediment cover than the other surveyed regions (Figure 3a). The NS and ZS transects demonstrated elevated estimates of combined biomass carbon (CBC, i.e., the combined carbon reservoir of reef fish and corals), coral carbon content (CC), and coral cover. Some NS transects excelled in terms of the fish assemblage carbon content. In general, the XS transects displayed lower values across most metrics than those in NS and ZS; however, these values were higher than those observed in the coastal areas.

**FIGURE 4:**
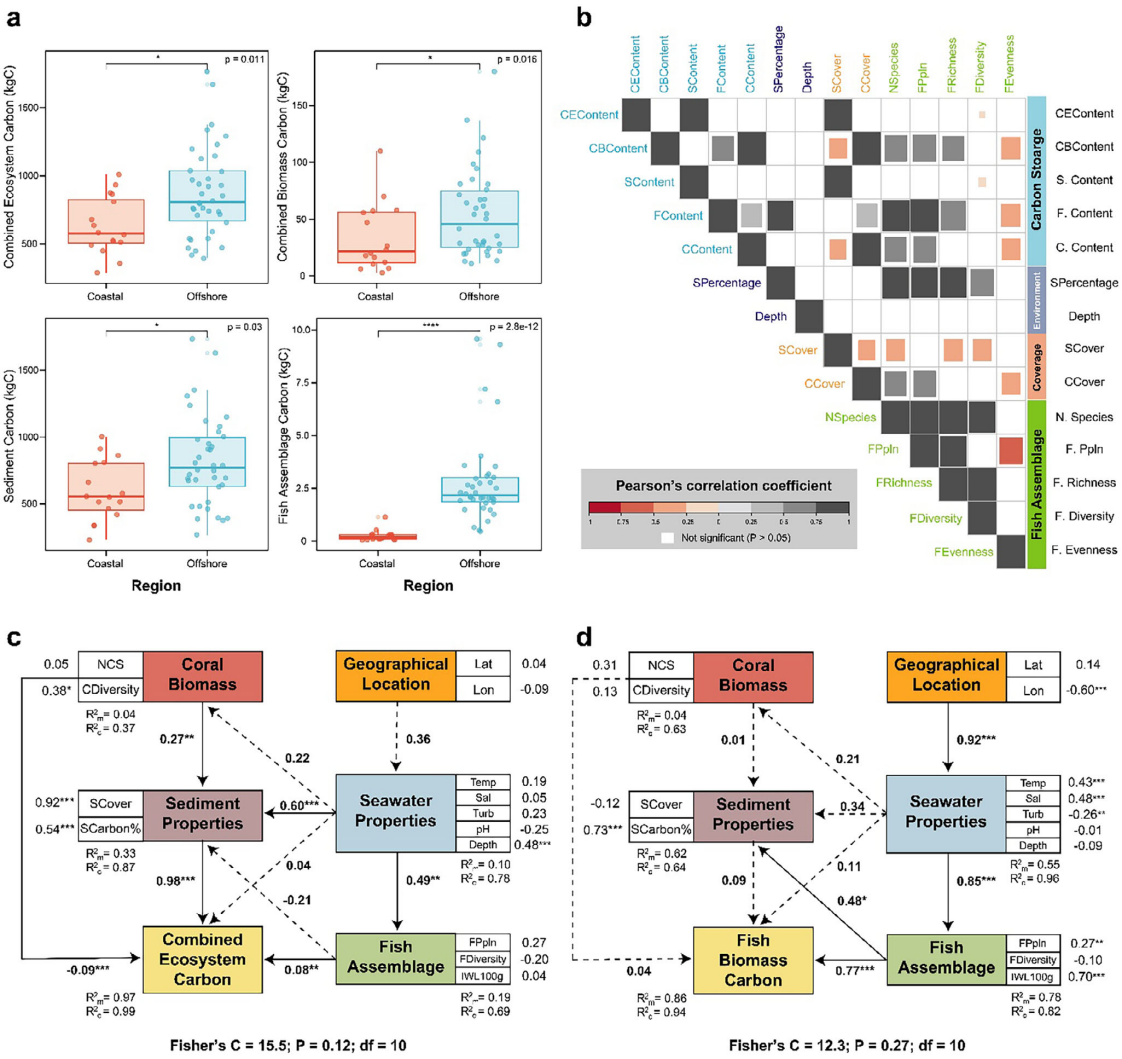
Correlations and piecewise structural equation models (pSEMs) for corresponding environmental and biological factors with total carbon reservoirs. (a) Comparison of carbon estimations between offshore (Nansha, Xisha, and Zhongsha Islands) and coastal (Guangdong and Hainan) regions. (b) Heatmap showing pairwise comparisons of transect properties using Pearson's correlation (only significant values are shown), as determined using the two‐sided cor.test in R. Properties are colored by category (carbon storage in blue, environment in purple, coverage in orange, and fish assemblage in green). The black and red squares represent positive and negative correlations, respectively. (c, d) pSEMs indicating the interplay of environmental and biological factors with combined ecosystem carbon (CEC) and fish biomass carbon (FC). Reef is considered a random factor. Seawater, sediment, coral, and fish assemblage factors (fixed factors) are divided into composite variables. Numerical values adjacent to the arrows represent path coefficients, indicating the standardized direct effect sizes of the relationships. Values adjacent to the measured variables (uncolored boxes) denote their ANOVA coefficients with CEC (c) and FC in (d). The solid lines represent statistically significant relationships, whereas the dashed lines represent insignificant paths. Statistically significant differences are denoted with * (^***^: *p* < 0.001, ^**^: *p* < 0.01, ^*^: *p* < 0.05). $R_{m}^{2}$ represents the percentage of marginal (explained by fixed factors) changes explained by pSEM. $R_{c}^{2}$ represents the percentage of marginal and random (explained by fixed and random factors) changes explained by pSEM.

The majority of the CECs were composed of carbon from the sediment (90.6%–95.7%, Figure 3b), similar to BCEs [36], where carbon was stored mainly in sediments and not in biomass. CEC tended to increase toward the equator, with GD at the lowest value and NS and ZS at the highest values. The portion of coral and fish carbon within the ecosystem also increased from higher to lower latitudes. The portion of CC increased from ∼5% in HN and GD to ∼9% in NS, and the portion of fish carbon increased from ∼0.03% to ∼0.5% (Figure 3b). CBC also increased further south (Figure 3c), with a more than twofold increase from ZS to NS. Corals contributed most of the carbon stocks in all the regions (93%–99%). An increasing trend in fish carbon contribution was observed with greater distance from the mainland (from 0.5% in the GD to 6% in the NS). With respect to the offshore (NS, XS, and ZS) and coastal (HN and GD) reefs, the offshore coral reef ecosystems had higher capacities in terms of CEC, CBC, SC, and FC (*p* = 0.011, 0.016, 0.03, and 2.8e^−12^, respectively) (Figure 4a).

Pairwise comparisons using the Pearson correlation revealed several prominent relationships across reef properties (Figure 4b). CEC and SC were positively correlated with sediment cover but negatively correlated with fish diversity. CBC increased with coral cover, fish population, number of species, and fish richness. Surprisingly, fish evenness was negatively correlated with CBC, FC, and CC. Thus, if a fish population is distributed evenly (i.e., if each species has a similar number of individuals) in a specific reef, then the overall carbon storage capacity can be undermined.

### 2.3 Impact of Fixed and Random Factors on Carbon Reservoirs

As illustrated in Figure 4c, pSEM can explain 97% of the marginal variance (attributable to fixed factors) and 2% of the conditional variance ($R_{c}^{2}-R_{m}^{2}$, attributable to random effects) in CEC. Therefore, seawater properties, sediment cover, coral cover, fish assemblages, and geographical location collectively account for the majority of the observed variation in coral reef carbon reservoirs, even in the presence of random effects associated with reefs at the sampling sites. Based on the pSEM results, CEC increased with sediment (0.98, *p* < 0.001) and fish population (0.08, *p* < 0.01) but decreased with coral biomass (−0.09, *p* < 0.001). Compared with fixed direct predictors ($R_{c}^{2}-R_{m}^{2}>R_{m}^{2}$), random factors (reef) can better explain seawater, sediment, coral, and fish assemblage composites. These measured factors are strongly correlated with the reefs where these coral reef ecosystems are located. Furthermore, pSEM explained 86% of the marginal changes (explained by fixed factors) and 8% of the random changes (explained by random factors) in FC (Figure 4d). Fish assemblage factors strongly influenced FC (0.77, *p* < 0.001). Conversely, coral and sediment factors have minimal and nonsignificant effects on fish carbon reservoirs. For all the VIFs of all fixed factors quantified, the absence of multicollinearity was ensured (VIFs < 10; Figure S5a).

### 2.4 Biological Mediations of the Coral Reef Carbon Budget

**FIGURE 5:**
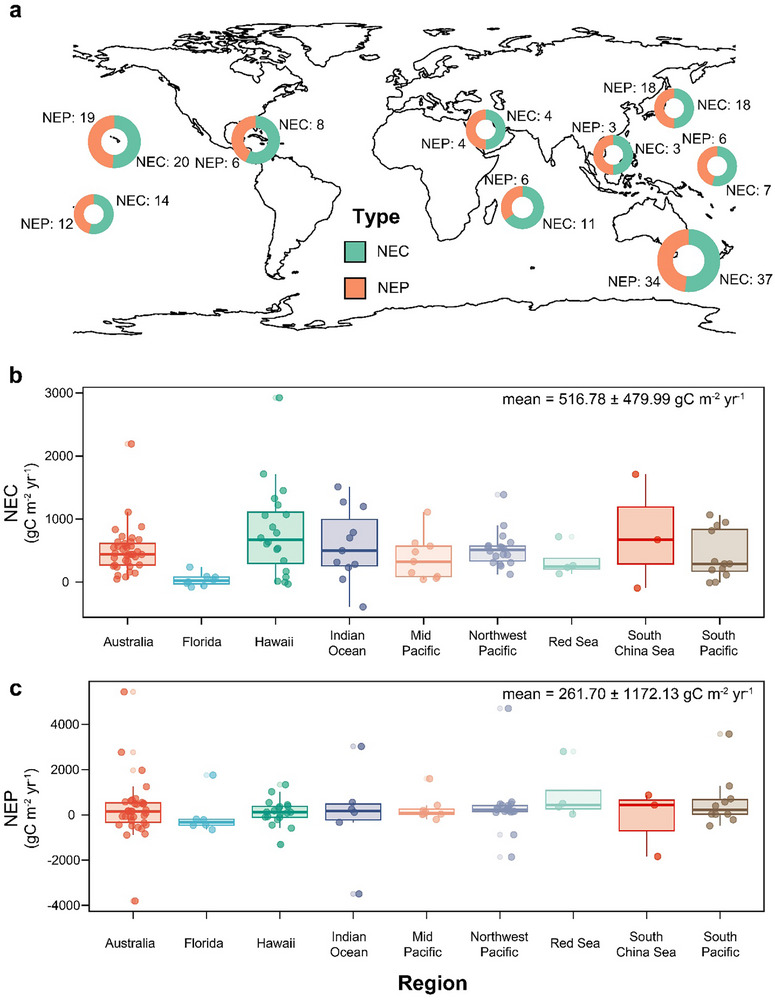
Net ecosystem calcification (NEC) and net ecosystem production (NEP) of coral reef ecosystems worldwide. (a) Number of NEC and NEP measurements recorded in different geographic regions taken from the literature review. The green portions represent the NEC, and the orange portions represent the NEP. (b, c) Box and dot plots indicating the NEC and NEP values in nine geographic regions of the world (Australia, Florida, Hawaii, the Indian Ocean, the Mid Pacific, the Northwest Pacific, the Red Sea, the SCS, and the South Pacific). The bars represent the minimum to maximum values of the NEC and NEP in each region. The horizontal line within the bar represents the median, and the whiskers represent the interquartile range.

On a broader level, NEC and NEP values were compiled from 58 published studies (1973–2025) spanning 14 countries/regions (Oceania: 4; Asia: 5; North America: 3; Africa: 2) (Figure 5a). NEC is defined as the net balance between calcification and dissolution from all calcifiers (corals, urchins, sponges, algae, fish, etc.), and NEP is defined as the net balance between photosynthesis and respiration [12, 37]. The synthesis included 41 reefs and atolls, yielding 122 NEC and 108 NEP estimates. The NEC ranged from −394.12 to 2924.09 gC m^−2^ yr^−1^ (mean = 516.78 ± 479.99 gC m^−2^ yr^−1^), with 22% of the reefs exhibiting net dissolution (NEC < 0) (Figure 5b). NEP ranged from −3803.69 to 5442.39 gC m^−2^ yr^−1^ (mean = 261.70 ± 1172.13 gC m^−2^ yr^−1^), with approximately 50% of the reefs in a state of net respiration (NEP < 0) (Figure 5c).

**FIGURE 6:**
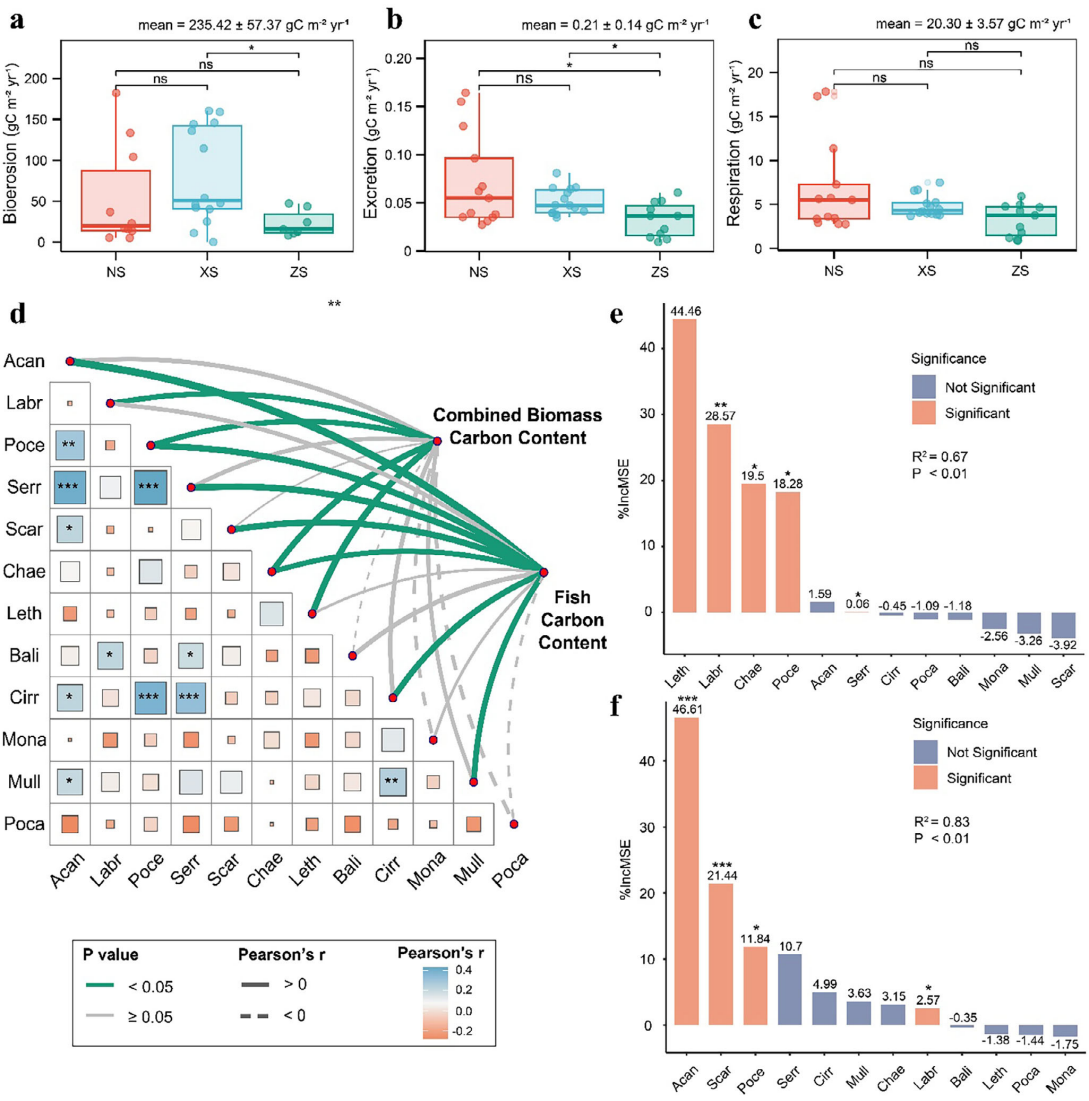
Impacts of reef fish assemblages on coral reef ecosystem carbon stocks. (a–c) Box plots of reef fish assemblage respiration, bioerosion, and excretion estimations of Nansha (NS), Xisha (XS), and Zhongsha (ZS). (d) Pearson correlations between carbon stocks, including combined biomass carbon (CBC) and fish assemblage carbon (FC), and populations of 12 reef fish families. The green lines represent statistically significant relationships between CBC and FC and the fish families. The solid lines and blue squares indicate positive relationships, whereas the dashed lines and orange squares indicate negative relationships. (e, f) Contributions of the main reef fish families driving changes in CBC and FC. Nonsignificant contributions are denoted in gray, and significant contributions are denoted in orange. Leth, Lethrinidae; Labr, Labridae; Chae, Chaetodontidae; Poce, Pomacentridae; Acan, Acanthuridae; Serr, Serranidae; Cirr, Cirrhitidae; Poca, Pomacanthidae; Bali, Balistidae; Mona, Monacanthidae; Mull, Mullidae; Scar, Scaridae. ^*^ *p* < 0.05, ^**^ *p* < 0.01, ^***^ *p* < 0.001.

Carbon fluxes were quantified via three primary pathways, namely, bioerosion, excretion, and respiration, and the contribution of reef fish assemblages to the coral reef carbon budget was assessed. Bioerosion contributed the greatest flux, with an average of 235.42 ± 57.37 gC m^−2^ yr^−1^ transferred from reef structures into the SC reservoir. The highest rates of bioerosion were recorded in XS (321.71 gC m^−2^ yr^−1^), followed by NS (214.60 gC m^−2^ yr^−1^) and ZS (92.60 gC m^−2^ yr^−1^) (Figure 6a). Ten species from the genera *Scarus* and *Chlorurus* were identified as bioeroders and incorporated into these estimates. Excretion by reef fish provided a relatively minor input, with 0.21 ± 0.14 gC m^−2^ yr^−1^ added to the sediment reservoir via the precipitation of ichthyocarbonates. Regional contributions were greatest in NS (0.29 gC m^−2^ yr^−1^), followed by XS (0.21 gC m^−2^ yr^−1^) and ZS (0.13 gC m^−2^ yr^−1^) (Figure 6b). Respiration accounted for an average release of 20.30 ± 3.57 gC m^−2^ yr^−1^ into the water column from fish metabolic processes. Respiration rates were broadly consistent across offshore regions, with the highest occurring in XS (23.01 gC m^−2^ yr^−1^), followed by NS (21.45 gC m^−2^ yr^−1^) and ZS (20.76 gC m^−2^ yr^−1^) (Figure 6c). Larger‐bodied fish exhibited disproportionately higher total CO_2_ production but lower per‐kilogram oxygen depletion (Figure S6). As parrotfish were rarely found in the coastal reefs of GD and HN, fish community data from these regions were excluded from assemblage‐level carbon budget analyses.

### 2.5 Relationships Between Reef Fish Families and Reef Carbon Stocks

As mentioned previously, only a few coral fish species were observed in coastal regions (HN and GD), and their fish species compositions differed significantly from those in offshore regions (NS, XS, and ZS) in the SCS region (Figure 2a). Therefore, fish community data from the HN and GD were excluded from the subsequent analysis. Pearson correlation analysis revealed significant positive correlations between CBC and the families Labridae, Pomacentridae, Chaetodontidae, and Lethrinidae (Figure 6d). In contrast, when the focus was solely on the carbon stock of the fish assemblages (FC), positive correlations were observed with Acanthuridae, Pomacentridae, Serranidae, Scaridae, Chaetodontidae, Cirrhitidae, and Mullidae (Figure 6d). No significant relationships were detected between Balistidae, Monacanthidae, or Pomacanthidae and either CBC or FC. Among the fish families, Serranidae was positively correlated with Acanthuridae, Cirrhitidae, and Pomacentridae, whereas Cirrhitidae was strongly and positively correlated with only Pomacentridae. The multicollinearity of the model revealed that all the VIFs were smaller than 4 (Figure S5b). Hierarchical variance partitioning analysis for the 12 coral reef fish families revealed that Lethrinidae, Labridae, Chaetodontidae, and Pomacentridae were the four most important fish families and explained 44.46%, 28.57%, 19.5%, and 18.28% of the variance in CBC, respectively (Figure 6e). The contribution of Serranidae was also statistically significant but accounted for only a very small amount of the variance (0.06%). The other seven fish families (Acanthuridae, Cirrhitidae, Pomacanthidae, Balistidae, Monacanthidae, Mullidae, and Scaridae) all had nonsignificant effects on CBC (Figure 6e). With respect to FC, Acanthuridae (46.61%), Scaridae (21.44%), Pomacentridae (11.84%), and Labridae (2.57%) contributed significantly (Figure 6f).

## 3 Discussion

In terms of the evaluation of marine ecosystem carbon reservoirs, previous studies have focused predominantly on BCEs, such as mangroves, salt marshes, and seagrass beds [38, 39]. Compared with BCEs, the coral reef ecosystems in the SCS region (the subject of this research) have immense potential for carbon storage. Accurate assessments of carbon storage are limited by the highly precise distribution area of coral reefs in the SCS. However, the most conservative estimate (25.73 TgC) still exceeds the total SC stock of all salt marshes in China (24.86 TgC, based on the assessment of Wang et al. [40]). Coral reef sediments represent the ultimate form of carbon sequestration on the seabed. More than 90% of the carbon in coral reef ecosystems is retained in sediments (SC), with corals and reef‐dwelling fish assemblages collectively contributing less than 10% (FC and CC). On a regional scale, compared with HN and GD in high‐latitude regions, reefs located farther from the mainland, such as NS, ZS, and XS in low‐latitude regions, have wider coverage of corals and possess greater carbon reservoirs (Figure 2b) [41, 42]. The differences in carbon reservoirs (CEC) among different coral reef areas are attributed mainly to the variations in the area of sedimentary zones.

The dynamic balance between carbon storage and release in coral reefs is influenced by multiple complex biological and nonbiological factors [43]. On the one hand, all living organisms are involved in regulating changes in coral reef structure (calcification and biological erosion) and metabolic changes (photosynthetic phosphorylation, respiration, and substance production) to varying degrees, influencing the dynamic balance between carbon storage and release [37, 44] (Figures 4 and 5). On the other hand, this dynamic balance is directly influenced by nonbiological factors, such as temperature, ocean currents, and air currents, on carbon dioxide dissolution processes in seawater [45] and indirectly affected by global changes and human interference in regulating coral reef communities and their biological processes [14, 46]. Previous studies have extensively reported highly variable NEPs and NECs in different coral reef areas around the world (Data S1). Considering the regional differences in coral reef distribution and the role of the combined effects of abiotic and biotic factors, the global research data can be weighted and averaged when the total NEP + NEC of global coral reefs is estimated to be 778.48 gC m^−2^ yr^−1^ (Figure 5b,c). If this approach is applied to the SCS region, then this average NEC + NEP of coral reefs is equivalent to 24.2% of the estimated average CEC (3215.80 gC m^−2^). However, this estimation is overly simplistic and fails to consider the various complex biological and nonbiological processes within the coral reef ecosystem. The findings of this study offer important information; that is, in most cases, coral reef ecosystems have a significant capacity for carbon sequestration, and organisms play crucial roles in these carbon cycling processes.

The findings of this research also clearly demonstrate that the fish and coral communities within coral reef ecosystems have considerable carbon storage potential. Fish population size (and thus total biomass/weight) and the number of fish species play critical roles in determining the magnitude of total biomass and fish carbon reservoirs in coral reefs (Figure 4b). The fish carbon stock calculated in this study (≈77.03 GgC) is equivalent to the seagrass meadow carbon stock of Liaoning and Guangxi (83.1 GgC) or the sum of the mangrove biomass carbon stock in Zhejiang, Fujian, Hong Kong, and Macau (79 GgC) [40]. However, the impact of reef‐dwelling biological communities on the carbon cycle of coral reef ecosystems far exceeds their direct biomass storage function. For example, one of the most important functions of coral reef fish species is to consume corals and algae, and they deposit the carbon they absorb onto the seabed for storage via the process of biological erosion [20, 47]. The estimates of this research reveal that reef fish communities transport an average of 235.63 gC m^−2^ yr^−1^ (bioerosion + excretion) into the SC pool via pathways that link CC and FC (Figure 6a,b). During these processes, carbon is first transferred from the CC into the FC (bioerosion), after which it partially passes on to the SC via excretion in the form of ichthyocarbonates. Compared with the average SC stock (3023.64 gC m^−2^), these fluxes amount to ∼7.8% per year, a contribution that far exceeds the static share of FC within CEC (0.03%–0.5%) (Figure 3b). This disproportionate influence not only underscores the critical role of fish assemblages in mediating SC dynamics but also highlights how animal communities amplify ecosystem‐scale carbon flux beyond their direct reservoirs. The magnitude of these contributions highlights a key ecological principle: conserving reef animal populations is not only critical for biodiversity but also essential for sustaining the pivotal role of sediments as long‐term carbon reservoirs in coral reef ecosystems.

In addition to their own carbon storage capacity (FC) and the regulation of carbon flux through biological erosion to influence CEC, the community assembly of coral reef fish species plays a crucial role in enhancing the stability of the ecosystem and the potential for biomass carbon storage (CBC). The ecological functions of coral reef fish species have been extensively studied because they directly shape reef community composition and function by grazing and predation [48]. As many fish species depend on living corals for food and shelter, rapid coral declines can reduce fish populations and biodiversity [49]. Furthermore, the abundance of fish communities benefits coral species recruitment and survivability via trophic cascades, such as herbivorous fish consumption of macroalgae that compete with corals [50] and predation on corallivorous crown‐of‐thorn starfish [51]. Transect records show that high population sizes of these coral fish species (e.g., Meiji Reef and Yongshu Reef in NS) tend to store more biomass carbon (CBC) than low population sizes (e.g., Manbu Ansha and Nanfei Ansha in ZS). Presumably, the extensive coral death caused by the proliferation of the crown‐of‐thorns starfish and destructive fishing in certain areas, which led to the uncontrolled regulation of coral fish populations, are the main factors contributing to the differences in CBC across different islands and reefs in the SCS region [52]. The findings of this study further demonstrate that certain families of coral reef fish species (Lethrinidae, Labridae, Chaetodontidae, and Pomacentridae) may serve as biological indicators of the carbon storage capacity of reef ecosystems in the SCS (Figure 6d–f). This positive cycle between coral and coral reef fish communities not only explains the relationship between coral reef biodiversity communities and CBC but also highlights the need to stabilize coral reef ecosystems, especially some key fish communities, for the future protection of coral reef carbon reservoirs.

In this research, carbon storage in the SCS region was estimated, and the contributions of corals and reef‐dwelling fish to carbon storage were systematically analyzed. Theoretically, although this method can be applied globally, a key challenge is the significant gaps in environmental and geographical data for global coral reef areas [27, 42, 53]. In particular, the uncertainty of the coral reef area contributed to discrepancies in the upper‐ and lower‐bound estimations of this research. Therefore, in addition to traditional field surveys, future research can combine optical remote sensing techniques, including the use of Landsat 8 and GF‐1 satellite images [54], to assess the global carbon storage of coral reefs. Furthermore, the findings of this research and a large number of previous studies highlight the need to elucidate how faunal assemblages influence carbon cycling processes in reef ecosystems [19, 25] and the need to quantify the carbon storage potential in coral reef frameworks [44, 55], benthic groups [56, 57], and plant species [23, 58]. More in‐depth future research is needed to identify not only which species are involved but also how these species influence carbon storage potential. These methods could provide valuable insights into conservation management in coral reef ecosystems. Finally, the allometric scaling of metabolic rates with body mass in reef fish species [59] should be considered in future research, as it can accurately estimate current fish respiration rates and predict future carbon flux changes under a fast‐changing climate [60].

Coral reef ecosystems are vast carbon reservoirs [15]. Reef‐dwelling organisms convert free carbon dioxide in the air and seawater into fixed carbon, which is then sequestered beneath the sea floor [11]. Coral reef ecosystems face increasing threats, such as rising sea surface temperatures, ocean acidification, sea‐level rise, overfishing, drilling, and dredging; amid these issues, these invaluable carbon reservoirs must be protected [61, 62]. Furthermore, although certain groups of coral and coral fish species can withstand climate change impacts, such as rising temperatures [23, 63], their overall carbon storage capacity is likely to deprecate. Regardless of whether coral reef ecosystems function as carbon sources or sinks, such degradation is likely to reduce carbon storage capacity and ultimately lead to the release of previously sequestered carbon into the ocean and atmosphere.

## 4 Conclusion

This study, which considered reefs in the SCS region as a representative case study, presents the comprehensive estimation of the CEC storage capacity of coral reef ecosystems. These findings underscore the substantial carbon storage potential of coral reef ecosystems and reveal significant differences in CEC and CBC between coastal and offshore reef habitats. Notably, the estimated CEC of SCS coral reefs surpasses that of all mangrove ecosystems in China and approaches the CEC of the nation's salt marshes. While sediment remains the dominant carbon pool within these systems, the results highlight the important role of animal taxa in modulating environmental and biomass carbon stocks. Additionally, specific reef fish families have strong predictive relationships with total biomass carbon and fish‐associated carbon pools, suggesting their potential utility as biological indicators for monitoring reef carbon dynamics. Furthermore, the results provide actionable insights into enhancing conservation prioritization and governance frameworks, emphasizing science‐based strategies to safeguard the biodiverse and climate‐vulnerable coral ecosystems of the SCS region. These findings align with emerging calls to integrate spatial planning and resilience metrics into marine management, offering a pathway to balance conservation objectives with sustainable development priorities in this geopolitically and ecologically dynamic seascape.

## 5 Experimental Section

### 5.1 Study Area

The study areas included 17 different coral reefs and coral communities in the SCS region, spanning from Nansha, Xisha, and Zhongsha Islands in the offshore to Hainan and Guangdong in coastal China (Figure 1a). Surveys were conducted in 2023 and 2024. The geographical coordinates of the study areas are listed in Table S2. The sites were preselected on the basis of previous studies of the coral ecosystems in the SCS to ensure representativeness [64, 65, 66, 67]. All survey sites were within a water depth of 20 m, mostly between 5 and 10 m, which are suitable growth ranges for most shallow‐water coral species [68].

### 5.2 Field Survey

Two divers conducted transects from a small boat to estimate the abundance of fish and coral species in the study area, and a stereo‐DOV system, belts, and quadrats were used. Three transect surveys (width of 5 m and length of 50 m) were conducted along the reef crest at each study location, each separated by at least 30 m (Figure 1b). Transect lengths were measured using a line reel. All transect surveys were performed during the day (8 am–5 pm). There were no changes in divers engaged in the surveys, and no equipment or methods were modified during the study period.

The stereo‐DOV system deployed at all survey locations was purchased from SeaGIS (Melbourne, Australia) and shipped to Guangzhou, China. The system consisted of an 80‐cm‐long aluminum bar with handles and two camera housings on each side (Figure 1c). Two GoPro Hero 9 video cameras, separated by 0.8 m with convergence angles of 8°, were mounted in each of the housings and were protected by aluminum brackets and waterproof covers. The camera resolution was set at 1440 (4:3 aspect ratio) and 60 fps to accommodate dynamic situations in the case of strong underwater turbulence. Each camera was calibrated prior to field surveys to account for light refraction and scattering effects when operating in the sea. The same cameras were used repeatedly in the left and right housings to prevent any instabilities in the calibration and minimize disturbance.

One diver operated the stereo‐DOV system and swam at a constant speed along the transect. The cameras were pointed forward and parallel to the belt. The video recording times ranged between 3 and 5 min depending on the substrate type and weather conditions. The distance between the diver and the reef was set to at least 70 cm to prevent camera vibrations and reduce perturbations to the fish communities [69]. Larger, more active fish species were surveyed on the first pass of each transect in a 5‐m‐wide belt, whereas the more territorial and abundant damselfish family was surveyed on a second pass of the transect in a 2‐m‐wide belt. Another diver swam ∼10 m behind the first diver and took benthic photos using a 50 m × 50 cm frame quadrat divided into 25 squares (10 m × 10 cm), which was randomly positioned on both sides of the transect every 5 m. Ten photos were taken for each transect. Each fish recorded during the surveys was identified to the lowest taxonomic level using morphological characteristics from the following publications and books: Coral Reef Fishes of the South China Sea: The Xisha, Nansha and Zhongsha Islands [70]; the Atlas of Marine Fishes’ Natural Color of the South China Sea [71]; and Marine Fishes of China [72]. The species identification results were also cross‐checked with FishBase [73] for the distribution, morphology, and habitat of each fish.

Reef fish and corals were collected for carbon percentage analysis. The fish samples were caught using handlines, gill nets, and underwater spearguns at the survey locations. All the participating personnel and equipment were consistent across all the surveys. A researcher conducted fishing activities with handlines on small boats simultaneously with transect surveys. Two gill nets were set at each survey location, each with a soak time (total time nets were in the water) of 2 h. Spearguns were operated by experienced divers near transects and aimed for larger species of fish (i.e., handline and gill nets tend to capture smaller species only). Live coral samples were collected using hammers and chisels. All the fish and coral samples were cleaned with fresh water in a boat and stored in −20°C refrigerators.

Two surface sediment samples were collected at each transect with a hand corer to account for the carbon reservoir in the benthic areas of the coral reefs. In this study, sediment is defined as the sandy portion of the benthic region in coral reefs, excluding the rocky and hard substrates, which contain only a minor fraction of carbon compared with the soft, sandy regions. On the basis of the average SCS coral reef lagoon sedimentation rate of 0.644 ± 0.14 cm yr^−1^ [74], surface sediments represent relatively young OC accumulation; thus, only the layer of the top 10 cm was collected. The sediment samples collected were mostly composed of sand, with exceedingly small fractions of rubble and gravel. The sediment cores were stored in −20°C refrigerators once retrieved.

### 5.3 Video and Photo Analysis

The video footage was first edited with Premiere Pro to include only the transect surveys, and they were further analyzed using EventMeasure software (SeaGIS). All the fish that passed through the field of view were recorded for their species, numbers, and lengths. To avoid double‐counting and overestimation of any roaming fish numbers, it was only considered a new observation when a fish species appeared at least 30 s after the last fish of the same species left the line of sight. All the fish samples were identified to the lowest possible taxonomic level. If a fish is more than 8 m away from the camera or if it is unrecognizable because of insufficient visibility or an elevated level of turbidity, then this specific fish is not recorded in the survey data. Furthermore, the total length of each encountered reef fish is measured only if its entire body (from the snout to the fork) is shown in the field of view. Many times during video recording, a large school of one fish species was captured by the camera; in this case, a selected number (10–15) of individual fish samples were measured, and their average lengths were set as the length for that species [75].

The coral cover and sediment cover of each survey location were estimated on the basis of the benthic photoquadrats analyzed in CoralNet online software (www.coralnet.ucsd.edu). Any duplicate photoquadrats were initially removed from the complete array of data to ensure that each transect had exactly 10 photos. The validated photos from each transect were subsequently examined in CoralNet to estimate the approximate coverage.

### 5.4 Fish Diversity and Biomass

Different R packages were used to analyze the species richness, diversity, and evenness of the fish communities along each transect. Species richness was calculated using the “margalef” function in the *abdiv* package, which does not simply measure the total number of species recorded but also the sampling size and effort. The biodiversity of the fish fauna was estimated using the “diversity” function in the *vegan* package, and the Shannon–Wiener index was used as the evaluation formula. Species evenness was also computed with the “evenness” function in the *asbio* package to consider ecosystem homogeneity in terms of the abundance of species.

The weight of each individual fish shown in the video footage was estimated using the length–weight relationship algorithm on FishBase [73]:

(1) $$W_{F}=a\;\times \;L^{b},$$

where W is the weight of the fish, a and b are constants specific to each fish species on FishBase, and L is the length of the fish. In cases where these coefficients are unavailable for a certain species, the constants of a congener with similar sizes and weights were used [69]. Given that no coefficients could be used for the entire genus of *Cirrhilabrus*, its weight was calculated from the average weight of five *Cirrhilabrus melanomarginatus* purchased at the local aquarium fish market in Hainan, China.

### 5.5 Carbon Stock Estimation

Previous research on fish carbon content has assumed the same fraction of carbon at 10%, 12.5%, and 15% for all species [76] or has used elemental analyzers to measure carbon levels [77]. In this research, all the coral fish species caught near the transects were assumed to have the same carbon percentage of 12.5% (OC+IC). However, data pertaining to the carbon percentages of coral species in the SCS remain limited. This issue was resolved by first washing and rinsing off any algae/sediment from the surfaces of the coral samples collected, and these samples were subsequently ground, dried, and combusted in a muffle furnace. The retrieved powder was used for OC and IC measurements. For OC, sample powders were combusted in a Shimadzu carbon analyzer (TOC‐L, automatic sample injector Shimadzu ASI‐L) at 950°C. Pretests were conducted using samples to stabilize the analyzer before actual estimations and ultimately avoid small errors associated with instrument variability. For IC, samples were tested using a Dietrich–Fruehling calcimeter, and the amount of carbon dioxide evolved from the reaction with hydrochloric acid (HCl) was estimated. Similar procedures were conducted for the sediment samples. The final carbon percentage was taken as the combination of OC and IC measurements.

The weight of each individual fish was subsequently multiplied by its carbon fraction to measure its carbon content. The total fish carbon stock of a transect was thus the sum of all the carbon biomass of every fish recorded in the stereo‐DOV videos. The value represents the reservoir for a 250 m^2^ (5 m × 50 m) area in that specific coral reef:

(2) $${\mathrm{CS}}_{F}=\sum_{i=1}^{n}\left(W_{F}\times P_{F}\right),$$

where CS_F_ is the fish biomass carbon stock for the entire transect, W_F_ is the weight of an individual fish, and P_F_ (12.5%) is the carbon percentage for the fish species.

For coral carbon estimation, a biomass conversion factor (F_C_) of 8.01 kg m^−2^ [78] was adopted when the weights of corals in a specific area, where the coral cover data were given, were analyzed. F_C_ was combined with the coral cover and coral carbon percentage to calculate the transect coral carbon stock:

(3) $${\mathrm{CS}}_{A}=\left(A_{C}\;\times \;250m^{2}\right)\;\times \;F_{C}\;\times \;P_{C},$$

where CS_A_ is the coral biomass carbon stock for the entire transect, A_C_ is the coral cover, F_C_ is the biomass conversion factor, and P_C_ is the carbon percentage of the SCS corals.

For sediment estimation, a density factor (D_S_) of 1.70 g cm^−3^ [79] was adopted when the weights of sediments in a specific transect were calculated. This factor was multiplied by the sediment area, sediment carbon percentage, and sediment thickness (0.1 m) to determine the transect sediment carbon stock:

(4) $${\mathrm{CS}}_{S}=\left(A_{S}\;\times \;250m^{2}\;\times \;0.1m\right)\;\times \;D_{S}\;\times \;P_{S},$$

where CS_S_ is the sediment carbon stock for the entire transect, A_S_ is the sediment cover, D_S_ is the sediment density, and P_S_ is the sediment carbon percentage.

The resulting carbon stocks of the fish, coral, and sediment samples collectively contribute to the total combined ecosystem carbon stock of the transect:

(5) $${\mathrm{CS}}_{T}={\mathrm{CS}}_{F}+{\mathrm{CS}}_{A}+{\mathrm{CS}}_{S},$$

where CS_T_ is the combined ecosystem carbon stock of a transect. The results were averaged to determine the transect stock of a particular region (e.g., all the transect stocks in the NSMJ and NSBWSZ were summed and averaged to represent the carbon reservoir of the Nansha Islands in a 250 m^2^ reef area).

Geographical data for the area of modern coral reefs in the SCS were collected from the literature to estimate the overall combined carbon reservoir for the region (Table S1, 80, 81, 82, 83). For accuracy, instead of multiplying the transect stock by the entire SCS reef area, the reef area data for each individual region (XS, ZS, NS, HN, and GD) were used. The relevant references are provided in Table S1. The combined ecosystem carbon reservoir in each area was calculated as follows:

(6) $$\mathrm{CEC}\;=\frac{A_{R}}{250m^{2}}\;\times CS_{T},$$

where CEC is the combined ecosystem carbon content in the region, A_R_ is the geographical area of the coral reefs, and CS_T_ is the average transect carbon stock of the region. For clarification, CEC is the summation of carbon stock in reef fish, corals, and surface sediment.

Similarly, only coral and fish assemblage reservoirs were included in the estimation of the combined biomass carbon content (CBC).

(7) $$\mathrm{CBC}\;=\frac{A_{R}}{250m^{2}}\times \left(CS_{F}+CS_{A}\right),$$

The sediment carbon content (SC), fish assemblage carbon content (FC), and coral carbon content (CC) were estimated as follows:

(8) $$\mathrm{SC}\;=\frac{A_{R}}{250m^{2}}\;\times CS_{T},$$

(9) $$\mathrm{FC}\;=\frac{A_{R}}{250m^{2}}\;\times CS_{F},$$

(10) $$\mathrm{CC}\;=\frac{A_{R}}{250m^{2}}\;\times CS_{A}.$$

### 5.6 Biological Mediations of the Coral Reef Carbon Budget

The potential influence of living organisms on the coral reef carbon reservoir was explored and quantified. The pathways in which reef fish taxa contribute to the overall carbon budget of the ecosystem were estimated from the following aspects: (a) bioerosion; (b) excretion; and (c) respiration. Ecosystem‐level biological processes driving CO_2_ changes were subsequently examined by measuring NEC and NEP.

With respect to fish bioerosion rates, the grazing effects of parrotfish assemblages across all surveyed transects in the SCS region were considered, and bioerosion was assumed to equal the rate of new sediment production by parrotfish [84, 85]. Parrotfish have long been considered a potential source of reef sediment and can produce large quantities of carbonate as a byproduct of grazing on reef surfaces [47, 86]. The annual amount of bioerosion caused by parrotfishes in each transect was calculated by multiplying the abundance (number of individuals) of each species by its specific erosion rate (kg CaCO_3_ m^−2^ yr^−1^). The data were taken from the works of Morgan and Kench [87] and Yarlett et al. [47].

With respect to fish excretion rates, this study applied the CaCO_3_ production rate–body mass relationship described by Salter et al. [88], who derived carbonate production rates (µmol CaCO_3_ h^−1^) from ichthyocarbonates excreted by 180 fish whose body masses ranged from 0.001 to 2.8 kg. This linear relationship was applied to all the fish recorded along the transects. Here, the sum of ichthyocarbonate production from transect‐level fish excretion was calculated. As parrotfishes excrete sediment (apart from feces) back into the environment as a byproduct of feeding [84, 87], the evaluation method applied in this study included only an estimated amount of regular feces for their excretion to avoid double‐counting carbon production.

With respect to fish respiration rates, live fish samples were first obtained in this research by using gill nets alongside the surveyed transects, targeting numerically dominant fish genera, such as *Scarus*, *Pomacentrus*, and *Chromis*. The collected fish samples were subsequently transferred to large containers with air stones and transported back to an indoor wet laboratory, where they were acclimatized to the new environment for 48 h. The water temperature was maintained at 25°C at an oxygen saturation of >90%. For each individual fish sample, oxygen consumption rates were measured using an intermittent flow respirometry system (Loligo Systems Tjele, Denmark) consisting of swim tunnels, waterflow controls, chambers, and measurement sensors. Background O_2_ consumption was measured in the respirometer for a minimum of three cycles before and after each set of trials to account for bacterial respiration. The resulting estimations were transformed to CO_2_ production rates during fish respiration and averaged for further calculations at the transect‐ and regional‐level respiration levels.

Apart from reef fishes, other organisms from the animal community impact the ecosystem carbon budget via two pathways: calcification and metabolism. NEC refers to the net balance between calcification and dissolution from all calcifiers (corals, urchins, sponges, or algae), whereas NEP refers to the net balance between photosynthesis and respiration [12, 37]. Carbonate dissolution and the uptake of DIC by photosynthesis reduce the *p*CO_2_ and increase the buffering capacity of seawater. However, calcification and respiration increase *p*CO_2_, which acts as a carbon source. These ecosystem‐level biological processes have been regarded as the principal drivers of the reef carbon budget [13, 14]. The relevant references concerning coral reef ecosystem calcification and metabolism are listed in the Data S1; the studies were searched on Web of Science and Google Scholar, with combinations of the terms “coral reef”, “calcification”, “metabolism”, “respiration”, “production”, and “carbon budget”. Studies were included in the estimation as long as the NEC/NEP measurements were documented.

### 5.7 Statistical Analysis

All data from the survey were log‐transformed and standardized to meet the linearity assumptions. The survey results of the fish population, diversity, richness, coral cover, sediment cover, and sediment carbon percentage in the coastal and offshore regions were subsequently compared, and the Wilcoxon test was applied to assess whether the collected data differed. Structural equation modeling (SEM), a robust statistical framework extensively used in ecological research [89, 90, 91], was applied to identify the factors influencing the magnitude of carbon reservoirs in coral reef ecosystems in the SCS region. SEM is particularly suited for large‐scale correlative studies because it can partition causal relationships among multiple interacting variables, thereby providing a comprehensive understanding of complex ecosystem processes. The piecewise structural equation modeling (pSEM) adopted in this study also allowed the incorporation of random factors, favoring datasets that are not normally distributed (as is the case in this research). Five factor categories, namely, seawater properties (sea surface temperature, salinity, turbidity, pH, and depth), sediment (sediment cover and sediment carbon percentage), coral (number of coral species and coral diversity), fish assemblage (fish population, fish diversity, and number of individuals with weight > 100 g), and geographical location (latitude and longitude), were selected as the fixed factors; in addition, the reef (listed as the sampling area in Table S2) was identified as the random factor. All observed variables were first divided into composite variables and subsequently included in the SEM. Multiple linear regressions were used to quantify the variance inflation factors (VIFs, a way to measure multicollinearity) at a threshold value of 10 [92, 93], thereby avoiding high correlation across the selected variables. Two pSEMs were constructed on the basis of the following hypothesis: (1) geographical location affects seawater properties; (2) seawater properties impact sediment, coral, and fish assemblages; (3) coral and fish assemblages impact sediment; and (4) seawater, sediment, coral, and fish assemblages affect the total carbon stock in reef ecosystems. pSEM tests were performed with the R package *piecewiseSEM*.

Finally, this study examined the relationships between carbon contents and the populations of the 12 most abundant reef fish families, including Lethrinidae, Labridae, Chaetodontidae, Pomacentridae, Acanthuridae, Serranidae, Cirrhitidae, Pomacanthidae, Balistidae, Monacanthidae, Mullidae, and Scaridae, to identify any reef fish families that are bioindicators of carbon reservoirs. The *Hmisc* and *linkET* packages in r were used to perform Pearson correlation analysis. The VIFs were quantified to avoid any multicollinearity across the fish families. The contributions of fish families to CBC (combined biomass carbon content) and FC (fish carbon content) were evaluated by performing hierarchical variance partitioning using the r package *glmm.hp* [94, 95]. All the statistical analyses were performed with R 4.4.2 [96, 97, 98, 99].

## Author Contributions

Y.C.: conceptualization; methodology; data curation; formal analysis; visualization; writing. W.Z.: conceptualization; methodology; investigation; resources; revision; supervision. F.W. and P.‐Y.Q.: conceptualization; funding acquisition; revision; supervision; project administration. L.Q.: visualization; investigation. H.L.: validation (species identification); investigation. M.H., Q.L., and W.Y.: investigation; resources (field surveys and sample collection).

## Conflicts of Interest

The authors declare no conflicts of interest.

## Supporting information




**Supporting File**: advs75285‐sup‐0001‐SuppMat.docx.

## Data Availability

The data that support the findings of this study are available from the corresponding author upon reasonable request.
