# Supplementary material for: Corals and Reef‐Dwelling Fish Regulate Carbon Storage and Cycling Processes in Coral Reef Ecosystems
Source: Adv Sci (Weinh). 2026 Apr 17;13(41):e20612. doi: 10.1002/advs.202520612 (PMC13335079; doi:10.1002/advs.202520612)
Supplement: Supplementary file 1 — Supporting File: advs75285‐sup‐0001‐SuppMat.docx. [file ADVS-13-e20612-s001.docx]

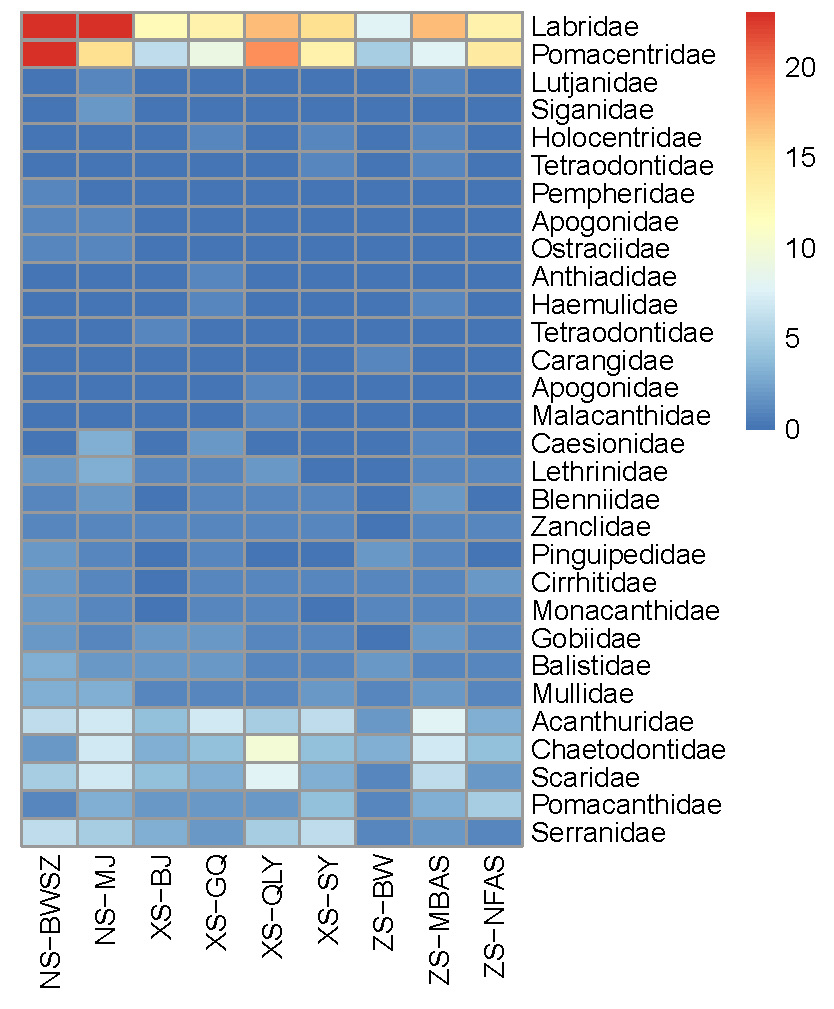


**S Fig. 1** Heatmap of species number of each family of the nine reefs in NS, ZS, and XS (excluding those in HN & GD, see Experimental Section in the main text for explanations).


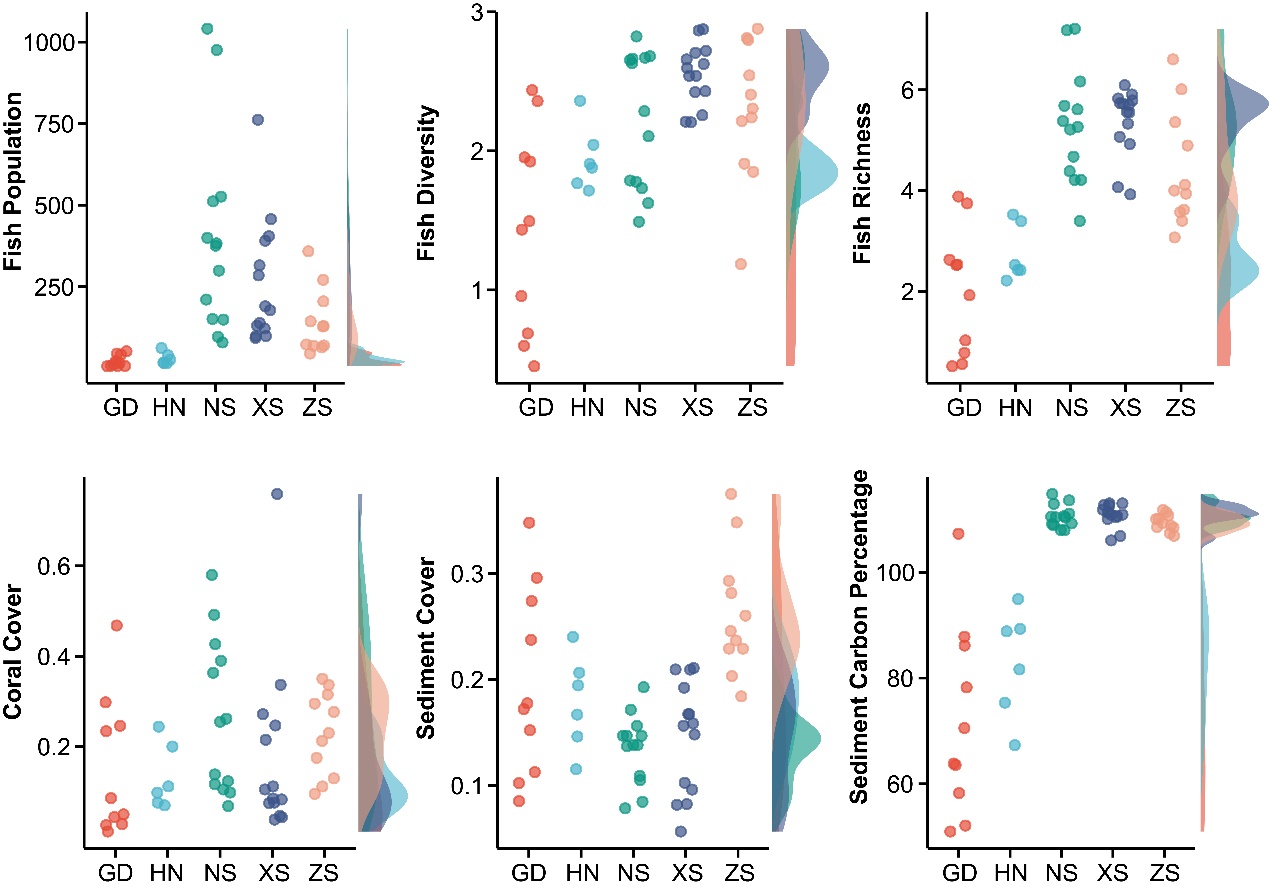


**S Fig. 2** Comparison of reef fish indexes in five different sampled regions (GD=Guangdong, HN=Hainan, NS=Nansha, XS=Xisha, ZS=Zhongsha).


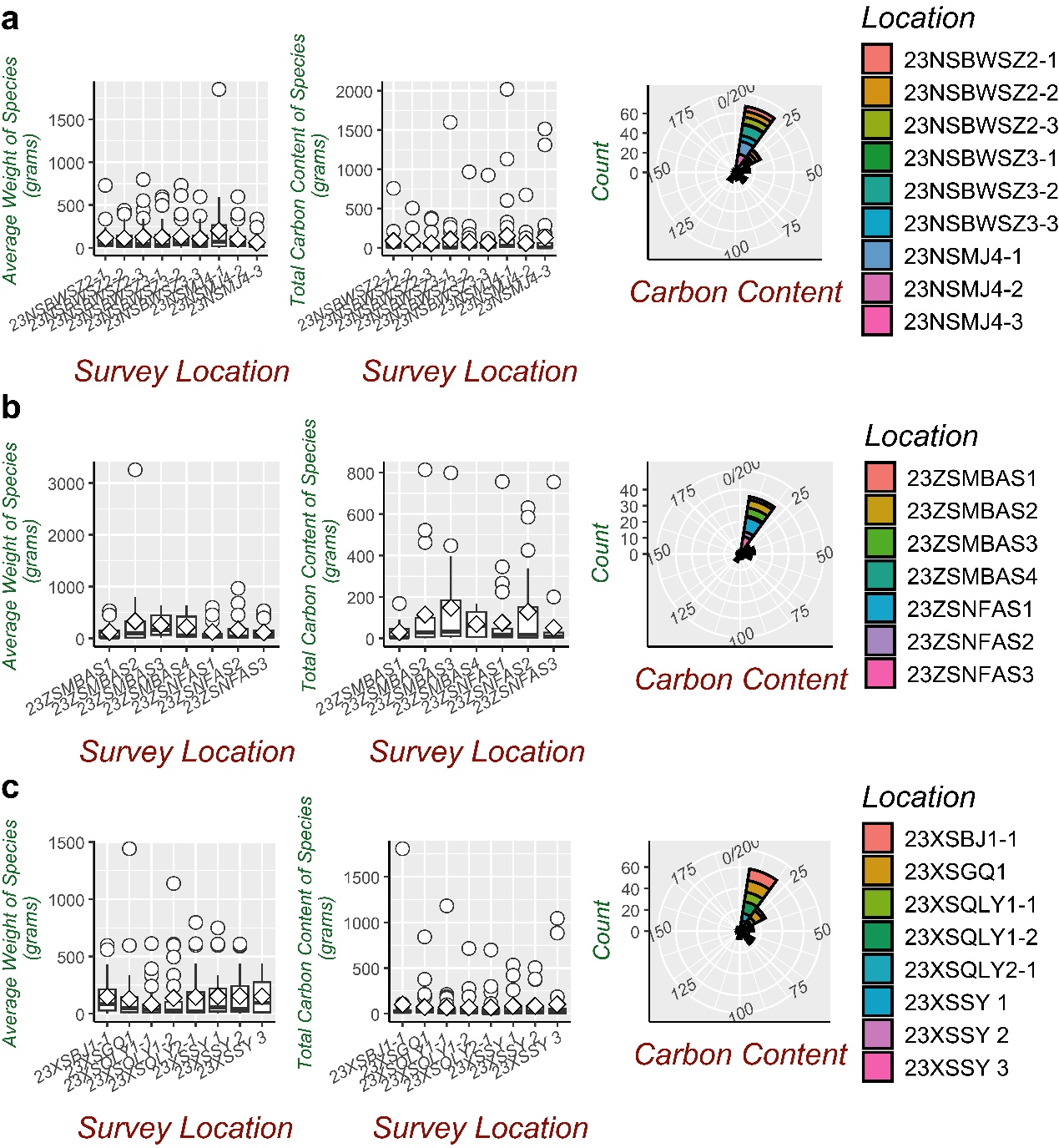


**S Fig. 3** The average weight and total carbon content of each fish genus in 2023 SCS cruise in **a** NS, **b** ZS, and **c** ZS.


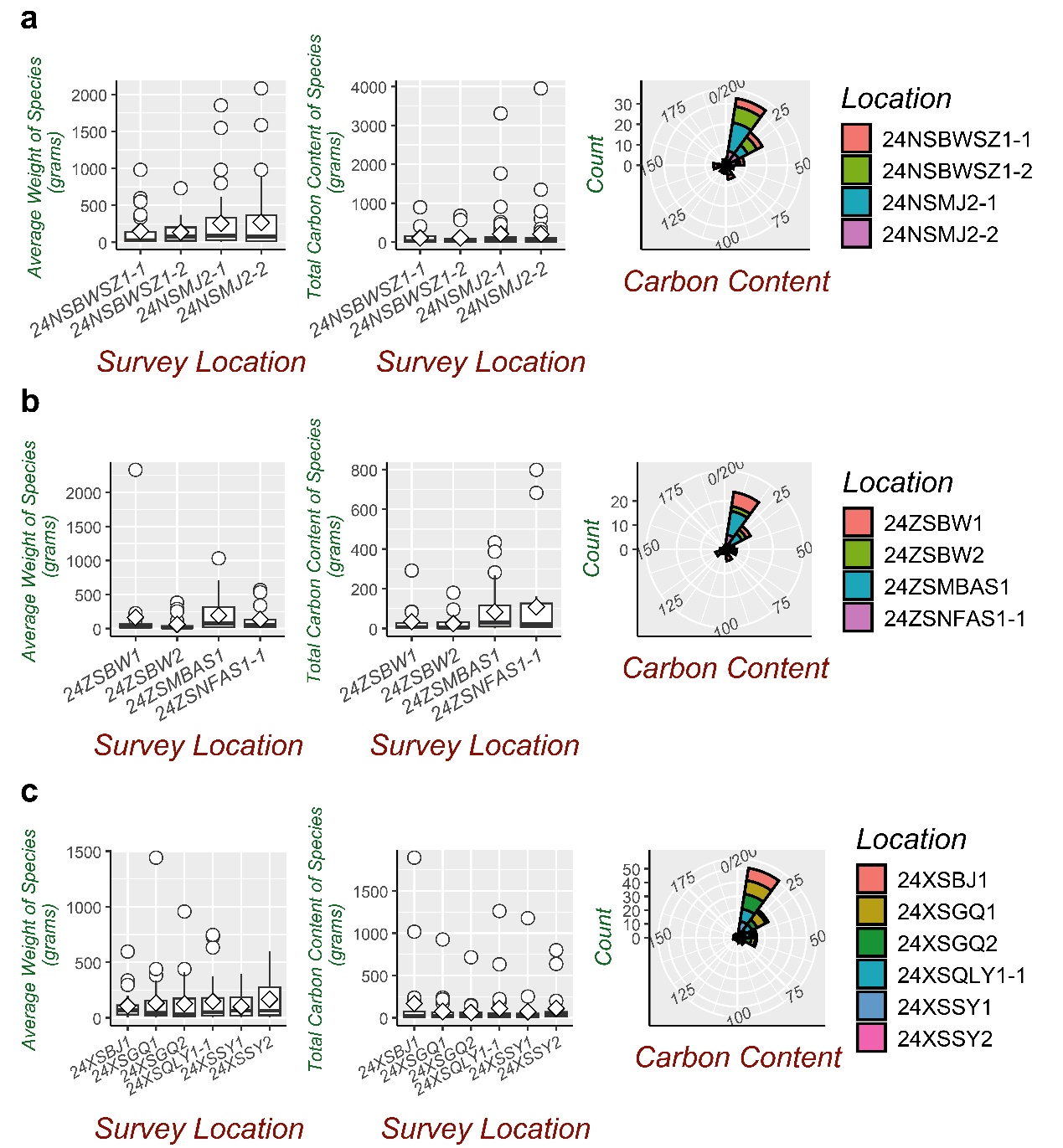


**S Fig. 4** The average weight and total carbon content of each fish genus in 2024 SCS cruise in **a** NS, **b** ZS, and **c** XS.


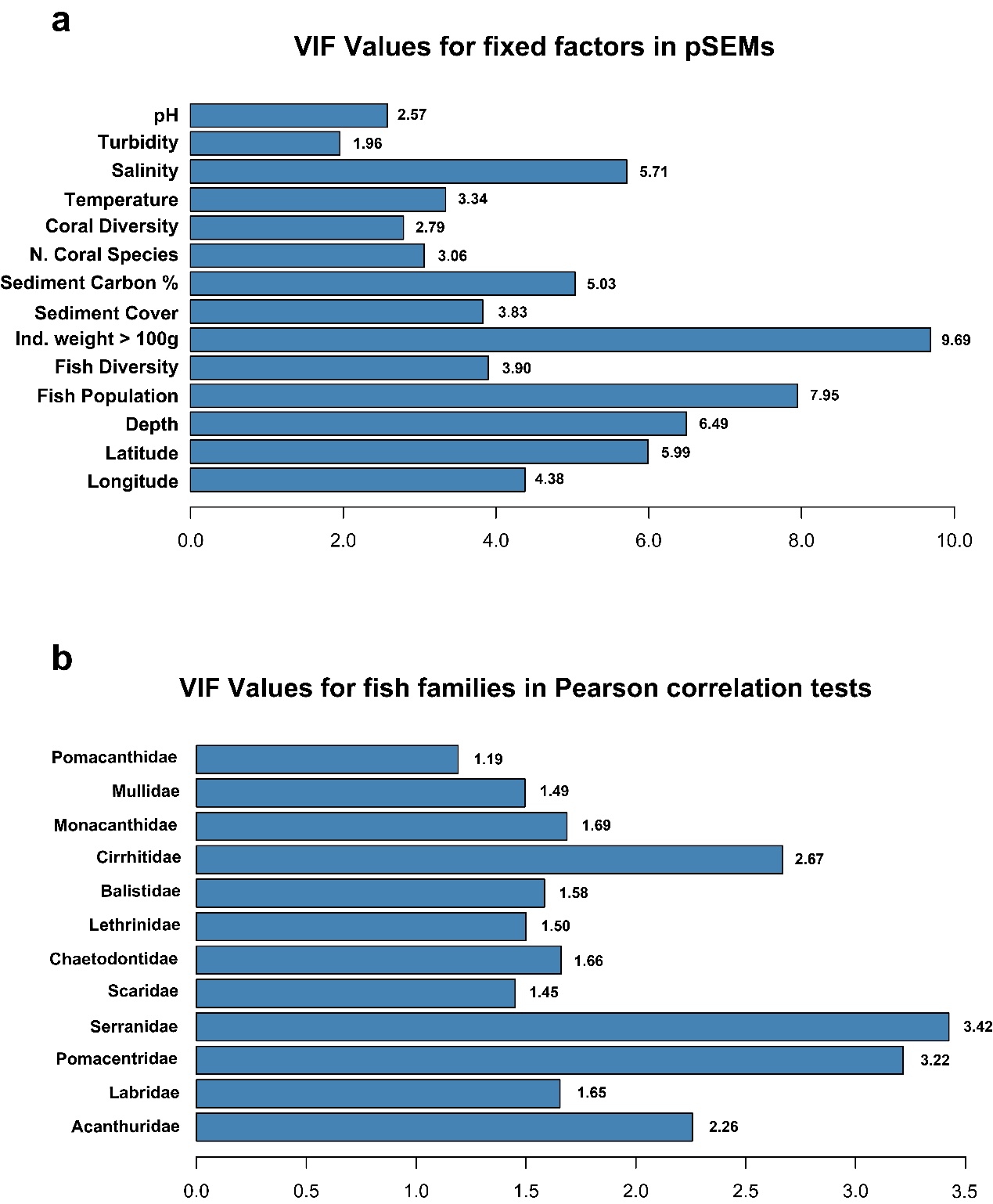


**S Fig. 5** Results of variance inflation factor (VIF) test for checking multicollinearity using multiple linear regression among the covariates in statistical analysis: a) bar plot showing VIFs for fixed factors used in pSEMs; b) bar plot showing VIFs for fish families used in Pearson correlation tests. Covariates with a value below 10 do not imply multicollinearity.


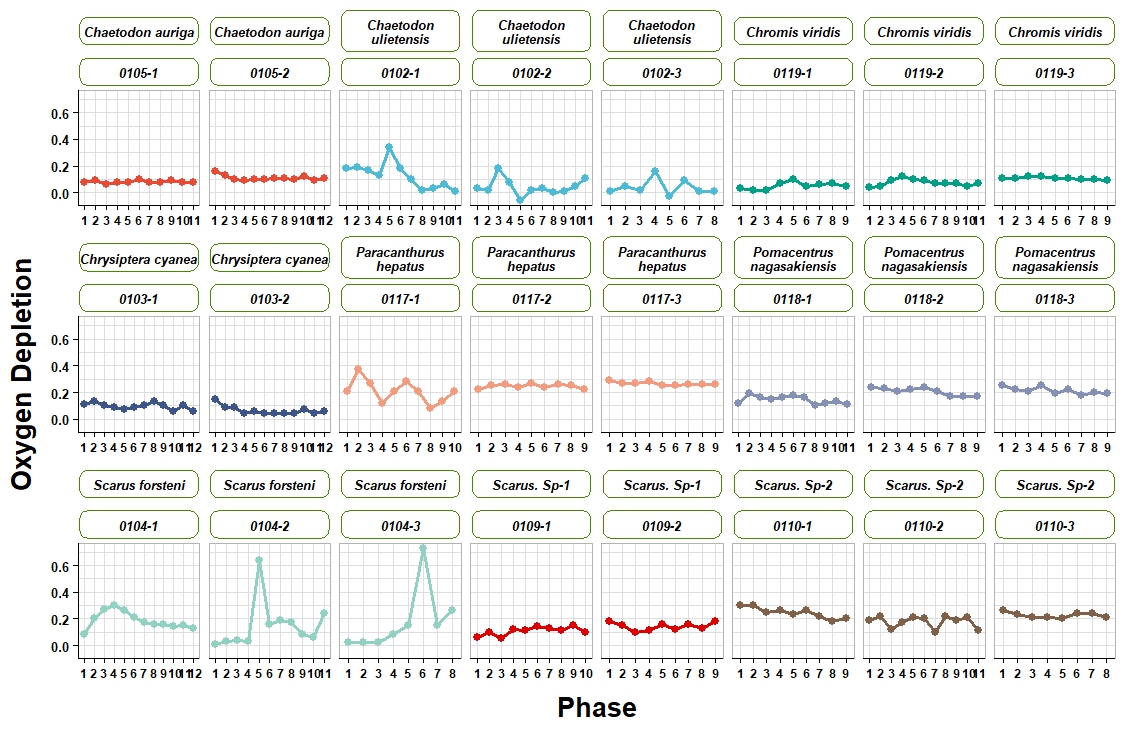


**S Fig. 6** Respiration rates (mg O2/kg/h) of fish species measured from the collected live fish samples from South China Sea. Data are colored by fish species & individuals. X axis is the trail phase and y axis is the oxygen depletion rate.


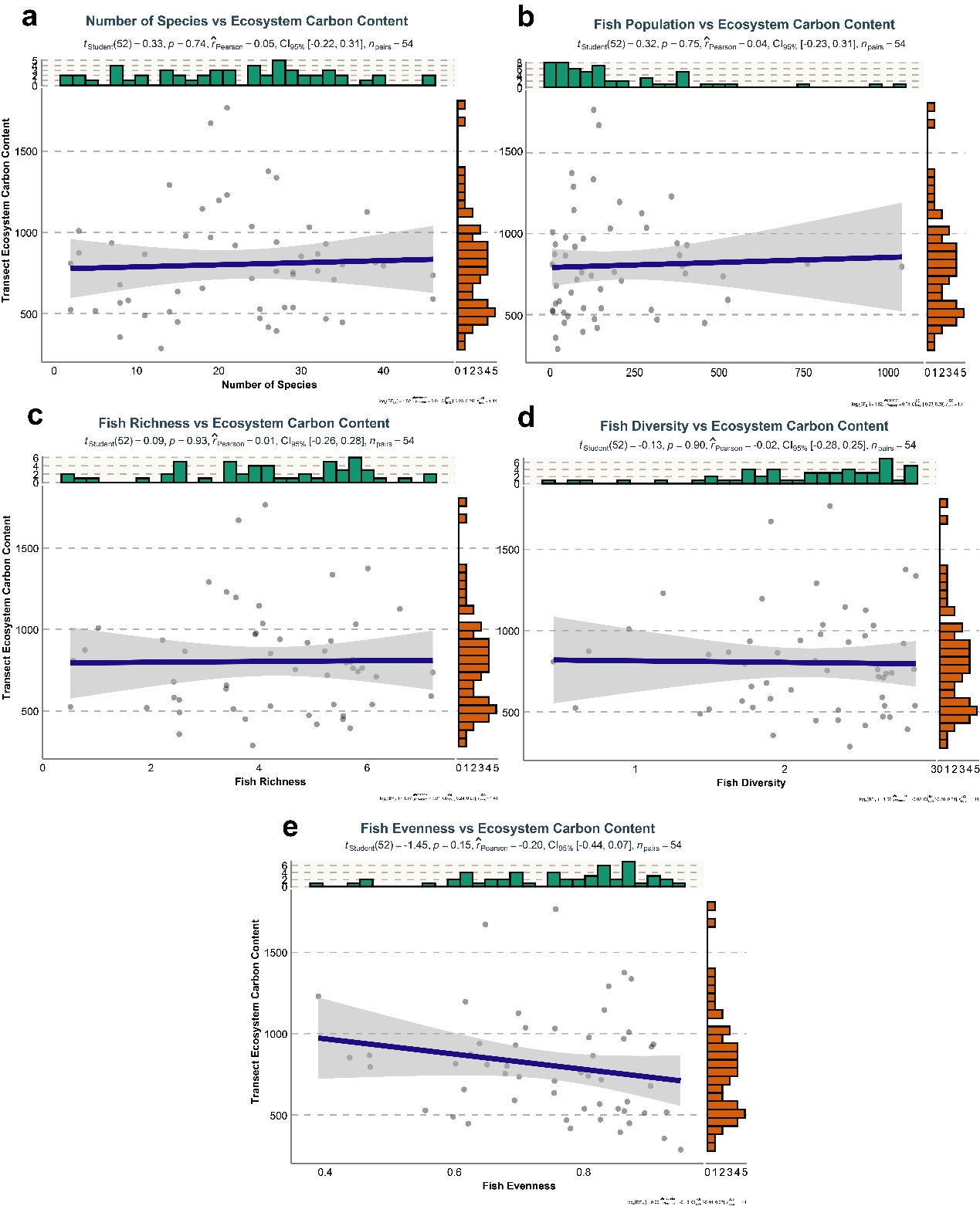


**S Fig. 7** Linear modeling for corelating five coral reef fish index to combined ecosystem carbon (CEC): **a** number of species, **b** population, **c** richness, **d** diversity, **e** evenness. Correlation, p-value, and confidence intervals are displayed for each linear model.


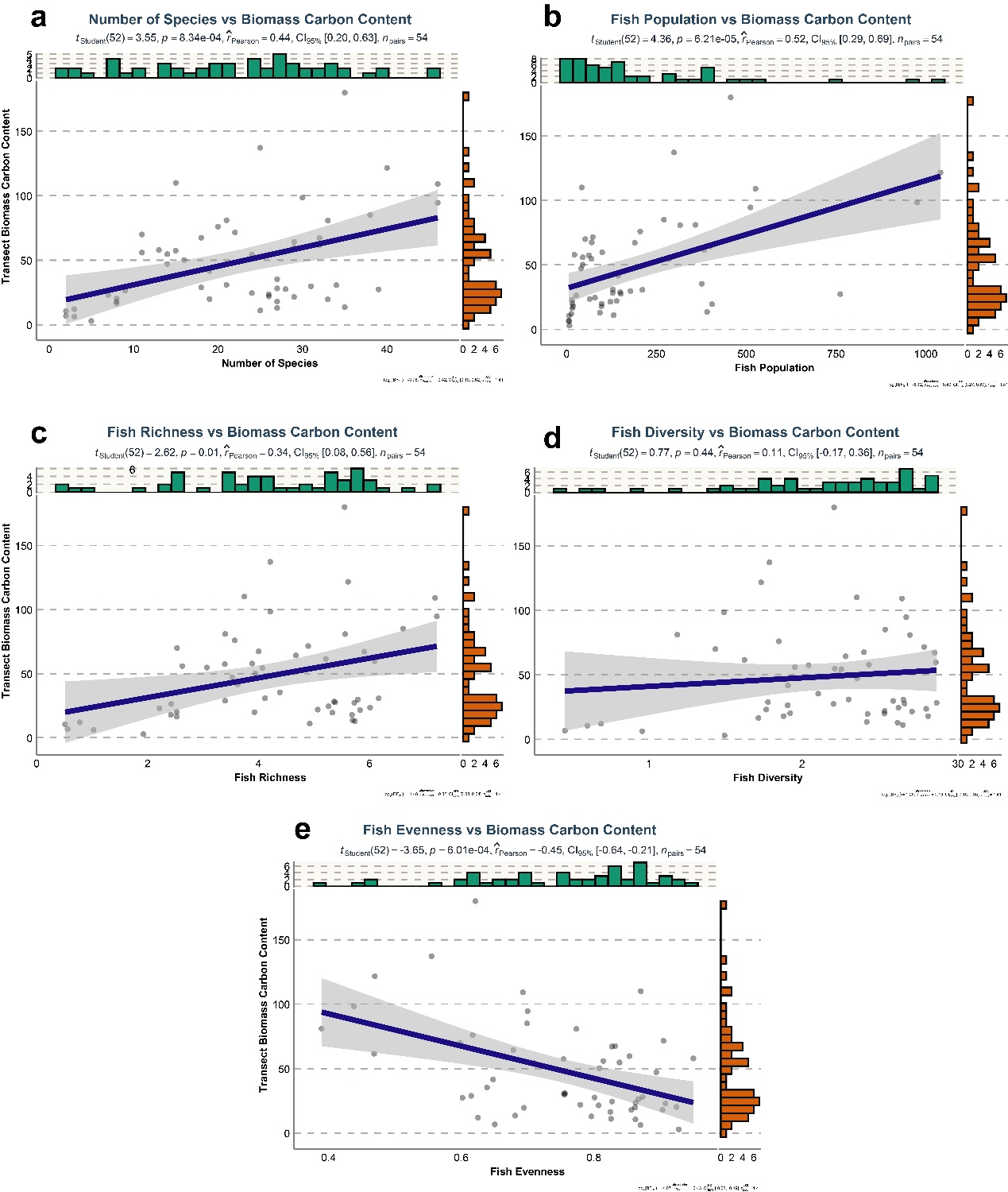


**S Fig. 8** Linear modeling for corelating five coral reef fish index to combined biomass carbon (CBC): **a** number of species, **b** population, **c** richness, **d** diversity, **e** evenness. Correlation, p-value, and confidence intervals are displayed for each linear model.


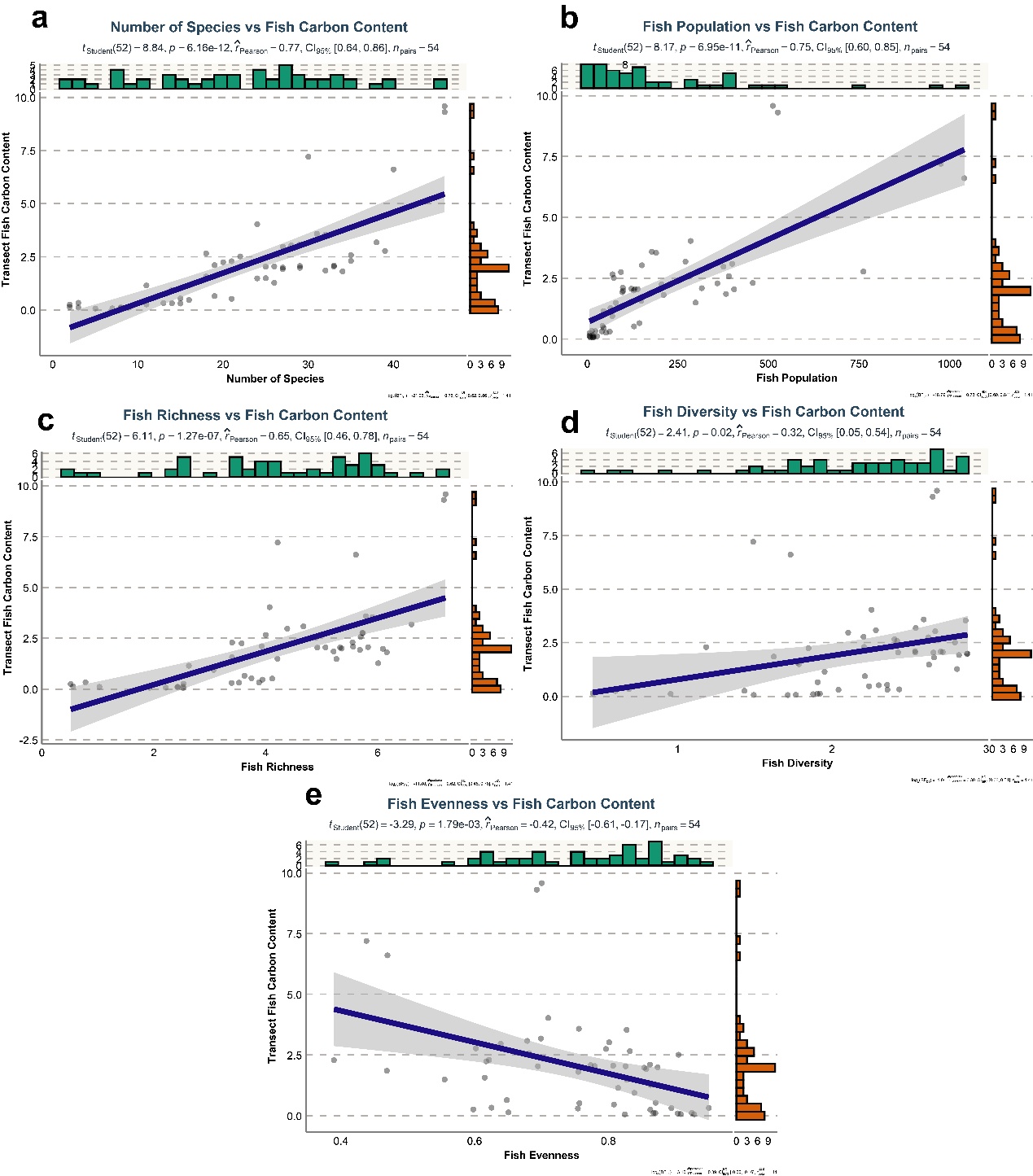


**S Fig. 9** Linear modeling for corelating five coral reef fish index to fish assemblage carbon (FC): **a** number of species, **b** population, **c** richness, **d** diversity, **e** evenness. Correlation, p-value, and confidence intervals are displayed for each linear model.

**S Table. 1** The average and combined ecosystem carbon (CEC) of coral reef ecosystems in SCS.


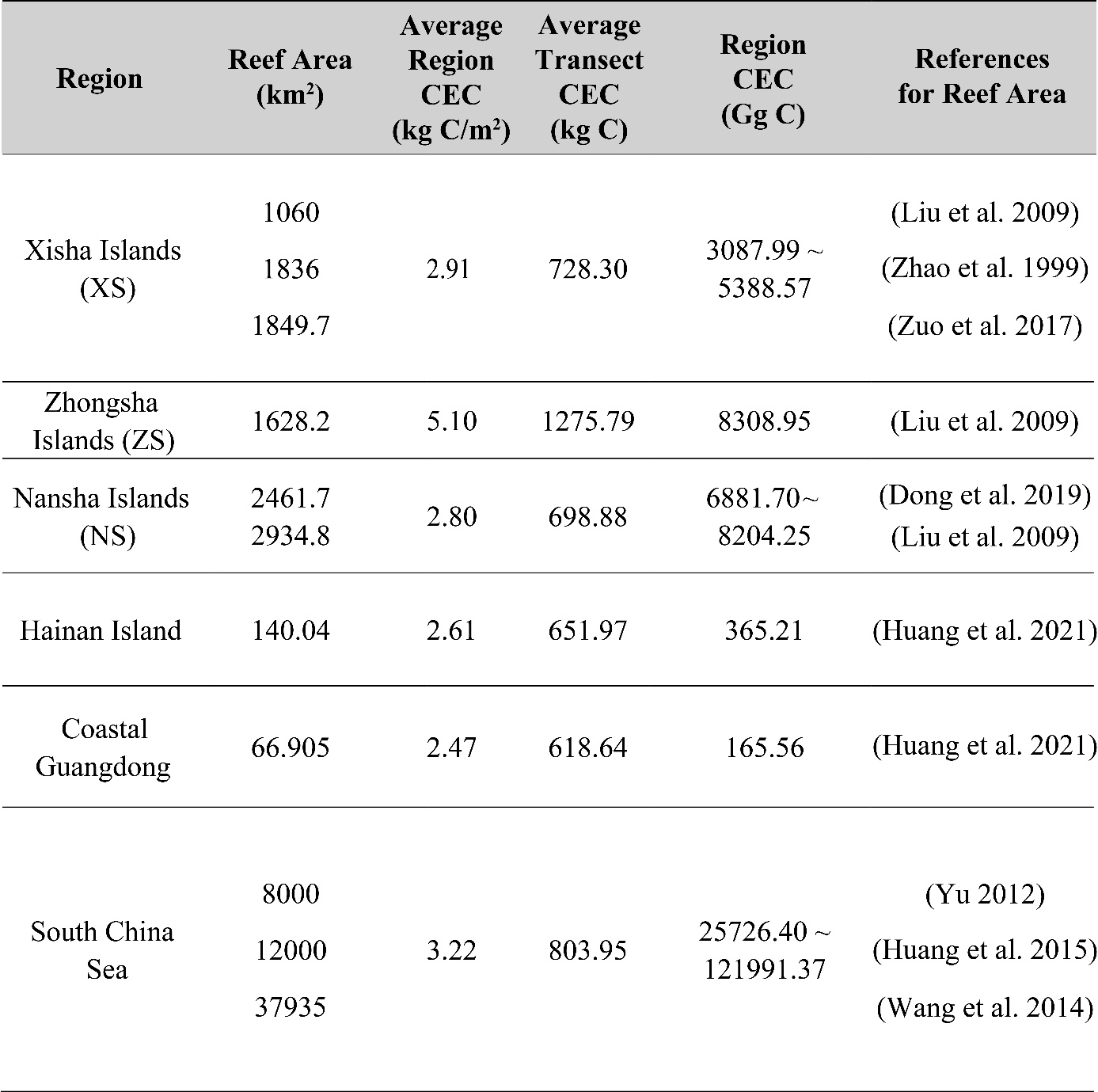


**S Table. 2** Sampling area, locations, and time in the SCS region


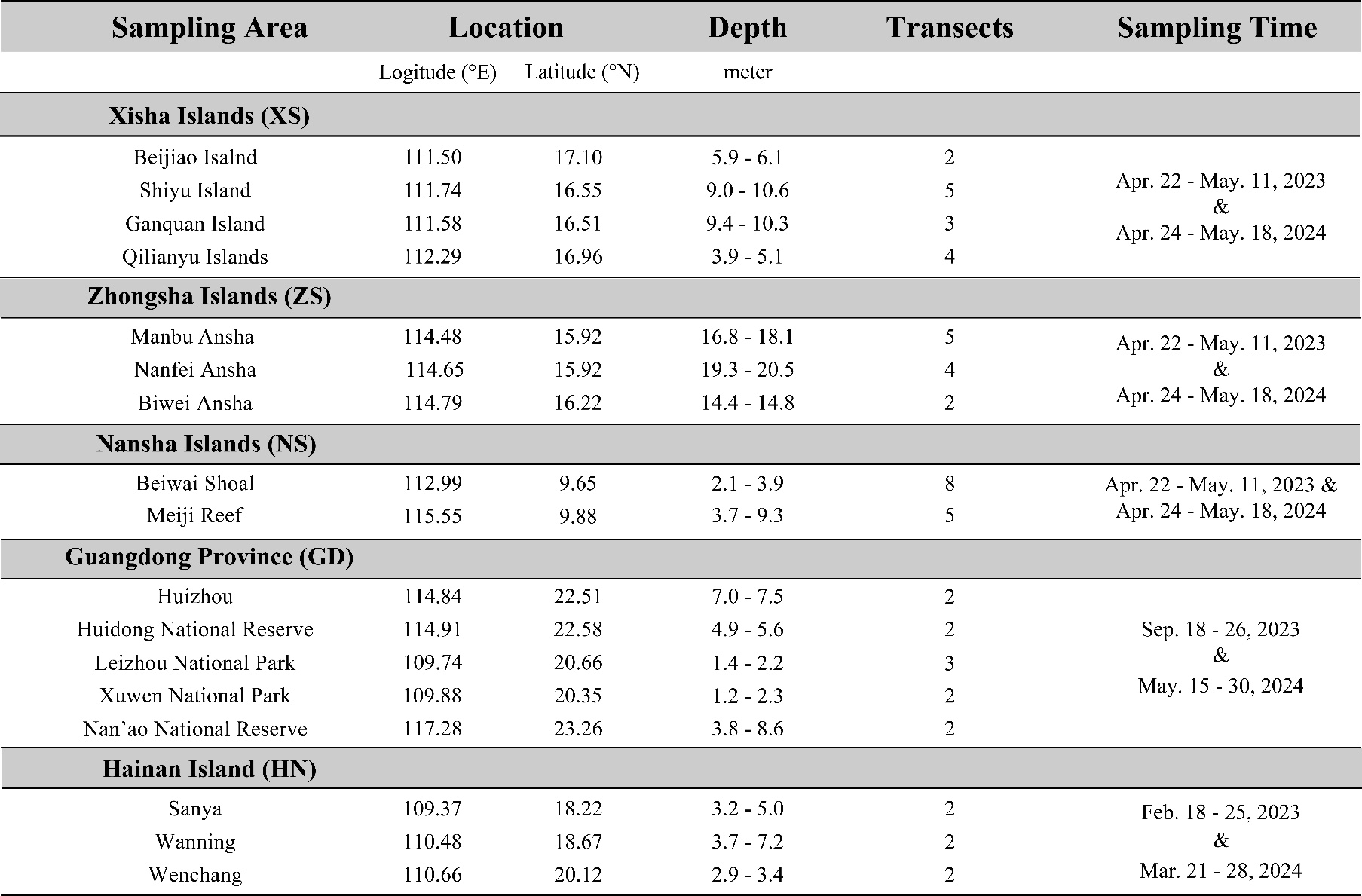


**S Table. 3** List of reef fish used for carbon content analysis

| **Family** | **Genus** | **Species** | **Sample Size** |
| --- | --- | --- | --- |
| Acanthuridae | Acanthurus | japonicus | 115 |
| Acanthuridae | Acanthurus | lineatus | 3 |
| Acanthuridae | Acanthurus | mata | 9 |
| Acanthuridae | Acanthurus | nigricauda | 2 |
| Acanthuridae | Acanthurus | nigrofuscus | 4 |
| Acanthuridae | Acanthurus | olivaceus | 9 |
| Acanthuridae | Acanthurus | pyroferus | 3 |
| Acanthuridae | Acanthurus | thompsoni | 2 |
| Acanthuridae | Ctenochaetus | binotatus | 167 |
| Acanthuridae | Ctenochaetus | cyanocheilus | 39 |
| Acanthuridae | Ctenochaetus | striatus | 778 |
| Acanthuridae | Naso | lituratus | 16 |
| Acanthuridae | Naso | minor | 9 |
| Acanthuridae | Paracanthurus | hepatus | 6 |
| Acanthuridae | Zebrasoma | scopas | 90 |
| Anthiadidae | Pseudanthias | dispar | 70 |
| Apogonidae | Apogonichthyoides | cathetogramma | 4 |
| Apogonidae | Cheilodipterus | quinquelineatus | 3 |
| Apogonidae | Ostorhinchus | angustatus | 27 |
| Apogonidae | Taeniamia | fucata | 3 |
| Balistidae | Melichthys | vidua | 41 |
| Balistidae | Rhinecanthus | rectangulus | 10 |
| Balistidae | Sufflamen | chrysopterum | 6 |
| Balistidae | Sufflamen | fraenatum | 1 |
| Balistidae | Xanthichthys | auromarginatus | 1 |
| Blenniidae | Meiacanthus | atrodorsalis | 1 |
| Blenniidae | Plagiotremus | rhinorhynchos | 11 |
| Blenniidae | Plagiotremus | tapeinosoma | 1 |
| Caesionidae | Caesio | lunaris | 4 |
| Caesionidae | Caesio | teres | 1214 |
| Caesionidae | Pterocaesio | tile | 40 |
| Carangidae | Caranx | melampygus | 1 |
| Centriscidae | Centriscus | scutatus | 1 |
| Chaetodontidae | Chaetodon | adiergastos | 1 |
| Chaetodontidae | Chaetodon | auriga | 2 |
| Chaetodontidae | Chaetodon | auripes | 3 |
| Chaetodontidae | Chaetodon | decussatus | 2 |
| Chaetodontidae | Chaetodon | kleinii | 47 |
| Chaetodontidae | Chaetodon | lunalatus | 8 |
| Chaetodontidae | Chaetodon | lunula | 1 |
| Chaetodontidae | Chaetodon | lunulatus | 16 |
| Chaetodontidae | Chaetodon | melannotus | 13 |
| Chaetodontidae | Chaetodon | modestus | 3 |
| Chaetodontidae | Chaetodon | octofasciatus | 1 |
| Chaetodontidae | Chaetodon | ornatissimus | 8 |
| Chaetodontidae | Chaetodon | punctatofasciatus | 15 |
| Chaetodontidae | Chaetodon | rafflesii | 2 |
| Chaetodontidae | Chaetodon | speculum | 4 |
| Chaetodontidae | Chaetodon | trifascialis | 18 |
| Chaetodontidae | Chaetodon | ulietensis | 4 |
| Chaetodontidae | Chaetodon | unimaculatus | 2 |
| Chaetodontidae | Chaetodon | vagabundus | 6 |
| Chaetodontidae | Chaetodon | xanthurus | 8 |
| Chaetodontidae | Forcipiger | longirostris | 4 |
| Chaetodontidae | Heniochus | chrysostomus | 3 |
| Cirrhitidae | Cirrhitichthys | falco | 2 |
| Cirrhitidae | Cirrhitus | pinnulatus | 7 |
| Cirrhitidae | Paracirrhites | arcatus | 57 |
| Cirrhitidae | Paracirrhites | forsteri | 1 |
| Fistulariidae | Fistularia | petimba | 2 |
| Gobiidae | Nemateleotris | magnifica | 109 |
| Gobiidae | Parioglossus | taeniatus | 1 |
| Gobiidae | Ptereleotris | evides | 23 |
| Gobiidae | Ptereleotris | zebra | 1 |
| Haemulidae | Plectorhinchus | polytaenia | 1 |
| Haemulidae | Plectorhinchus | vittatus | 1 |
| Holocentridae | Sargocentron | caudimaculatum | 5 |
| Labridae | Anampses | caeruleopunctatus | 3 |
| Labridae | Anampses | meleagrides | 2 |
| Labridae | Bodianus | anthioides | 3 |
| Labridae | Bodianus | axillaris | 8 |
| Labridae | Bodianus | bilunulatus | 1 |
| Labridae | Cheilinus | fasciatus | 3 |
| Labridae | Cheilinus | trilobatus | 15 |
| Labridae | Cirrhilabrus | cyanopleura | 2 |
| Labridae | Cirrhilabrus | melanomarginatus | 839 |
| Labridae | Coris | aygula | 1 |
| Labridae | Coris | gaimard | 9 |
| Labridae | Epibulus | brevis | 1 |
| Labridae | Epibulus | insidiator | 4 |
| Labridae | Gomphosus | varius | 44 |
| Labridae | Halichoeres | argus | 1 |
| Labridae | Halichoeres | biocellatus | 5 |
| Labridae | Halichoeres | hortulanus | 36 |
| Labridae | Halichoeres | margaritaceus | 4 |
| Labridae | Halichoeres | marginatus | 4 |
| Labridae | Halichoeres | nebulosus | 18 |
| Labridae | Halichoeres | nigrescens | 7 |
| Labridae | Halichoeres | tenuispinis | 1 |
| Labridae | Halichoeres | trimaculatus | 2 |
| Labridae | Hemigymnus | fasciatus | 11 |
| Labridae | Hemigymnus | melapterus | 6 |
| Labridae | Hologymnosus | annulatus | 4 |
| Labridae | Hologymnosus | doliatus | 2 |
| Labridae | Labrichthy | unilineatus | 22 |
| Labridae | Labroides | bicolor | 5 |
| Labridae | Labroides | dimidiatus | 74 |
| Labridae | Labroides | pectoralis | 6 |
| Labridae | Labropsis | xanthonota | 2 |
| Labridae | Macropharyngodon | meleagris | 7 |
| Labridae | Oxycheilinus | bimaculatus | 2 |
| Labridae | Oxycheilinus | digramma | 2 |
| Labridae | Oxycheilinus | unifasciatus | 42 |
| Labridae | Pseudocheilinus | evanidus | 5 |
| Labridae | Pseudocheilinus | hexataenia | 12 |
| Labridae | Pseudocheilinus | octotaenia | 17 |
| Labridae | Stethojulis | bandanensis | 13 |
| Labridae | Stethojulis | interrupta | 5 |
| Labridae | Thalassoma | amblycephalum | 862 |
| Labridae | Thalassoma | hardwicke | 56 |
| Labridae | Thalassoma | lunare | 14 |
| Labridae | Thalassoma | quinqueevittatum | 618 |
| Lethrinidae | Gnathodentex | aureolineatus | 375 |
| Lethrinidae | Lethrinus | nebulosus | 1 |
| Lethrinidae | Lethrinus | xanthochilus | 3 |
| Lethrinidae | Monotaxis | grandoculis | 3 |
| Lethrinidae | Monotaxis | heterodon | 2 |
| Lutjanidae | Aphareus | furca | 3 |
| Lutjanidae | Aprion | virescens | 2 |
| Malacanthidae | Malacanthus | brevirostris | 1 |
| Monacanthidae | Cantherhines | dumerilii | 1 |
| Monacanthidae | Cantherhines | dumerilii | 2 |
| Monacanthidae | Cantherhines | pardalis | 16 |
| Monacanthidae | Monacanthus | chinensis | 9 |
| Mullidae | Mulloidichthys | flavolineatus | 1 |
| Mullidae | Parupeneus | biaculeatus | 2 |
| Mullidae | Parupeneus | crassilabris | 3 |
| Mullidae | Parupeneus | cyclostomus | 2 |
| Mullidae | Parupeneus | multifasciatus | 24 |
| Mullidae | Parupeneus | pleurostigma | 1 |
| Nemipteridae | Pentapodus | setosus | 2 |
| Ostraciidae | Ostracion | cubicus | 1 |
| Ostraciidae | Ostracion | meleagris | 2 |
| Pempheridae | Pempheris | oualensis | 2 |
| Pempheridae | Pempheris | schwenkii | 5 |
| Pinguipedidae | Parapercis | clathrata | 9 |
| Pinguipedidae | Parapercis | hexophthalma | 1 |
| Pinguipedidae | Parapercis | millepunctata | 1 |
| Plotosidae | Plotosus | lineatus | 1 |
| Pomacanthidae | Apolemichthys | trimaculatus | 3 |
| Pomacanthidae | Centropyge | bispinosa | 3 |
| Pomacanthidae | Centropyge | heraldi | 48 |
| Pomacanthidae | Centropyge | tibicen | 6 |
| Pomacanthidae | Centropyge | vroliki | 68 |
| Pomacanthidae | Pygoplites | diacanthus | 5 |
| Pomacentridae | Abudefduf | bengalensis | 19 |
| Pomacentridae | Abudefduf | sexfasciatus | 14 |
| Pomacentridae | Abudefduf | vaigiensis | 31 |
| Pomacentridae | Amblyglyphidodon | curacao | 3 |
| Pomacentridae | Amphiprion | clarkii | 23 |
| Pomacentridae | Amphiprion | sandaracinos | 1 |
| Pomacentridae | Chromis | margaritifer | 527 |
| Pomacentridae | Chromis | notata | 40 |
| Pomacentridae | Chromis | ternatensis | 162 |
| Pomacentridae | Chromis | vanderbilti | 212 |
| Pomacentridae | Chromis | viridis | 49 |
| Pomacentridae | Chromis | xanthura | 9 |
| Pomacentridae | Chrysiptera | biocellata | 3 |
| Pomacentridae | Chrysiptera | brownriggii | 159 |
| Pomacentridae | Chrysiptera | chrysocephala | 8 |
| Pomacentridae | Chrysiptera | unimaculata | 6 |
| Pomacentridae | Dascyllus | reticulatus | 53 |
| Pomacentridae | Dascyllus | trimaculatus | 10 |
| Pomacentridae | Dischistodus | melanotus | 2 |
| Pomacentridae | Neoglyphidodon | crossi | 9 |
| Pomacentridae | Neoglyphidodon | nigroris | 4 |
| Pomacentridae | Neoglyphidodon | thoracotaeniatus | 13 |
| Pomacentridae | Neopomacentrus | bankieri | 23 |
| Pomacentridae | Neopomacentrus | cyanomos | 20 |
| Pomacentridae | Plectroglyphidodon | dickii | 79 |
| Pomacentridae | Plectroglyphidodon | lacrymatus | 162 |
| Pomacentridae | Plectroglyphidodon | leucozonus | 12 |
| Pomacentridae | Pomacentrus | alexanderae | 20 |
| Pomacentridae | Pomacentrus | amboinensis | 1 |
| Pomacentridae | Pomacentrus | bankanensis | 4 |
| Pomacentridae | Pomacentrus | brachialis | 8 |
| Pomacentridae | Pomacentrus | cheraphilus | 1 |
| Pomacentridae | Pomacentrus | chrysurus | 17 |
| Pomacentridae | Pomacentrus | coelestis | 118 |
| Pomacentridae | Pomacentrus | lepidogenys | 4 |
| Pomacentridae | Pomacentrus | moluccensis | 3 |
| Pomacentridae | Pomacentrus | nigromarginatus | 7 |
| Pomacentridae | Pomacentrus | pavo | 2 |
| Pomacentridae | Pomacentrus | philippinus | 93 |
| Pomacentridae | Pomacentrus | tripunctatus | 4 |
| Pomacentridae | Pomacentrus | vaiuli | 90 |
| Pomacentridae | Pycnochromis | atripes | 3 |
| Pomacentridae | Pycnochromis | margaritifer | 240 |
| Pomacentridae | Pycnochromis | ovatiformis | 5 |
| Pomacentridae | Pycnochromis | vanderbilti | 1292 |
| Pomacentridae | Stegastes | fasciolatus | 21 |
| Pomacentridae | Stegastes | insularis | 7 |
| Pomacentridae | Stegastes | obreptus | 60 |
| Scaridae | Chlorurus | microrhinos | 7 |
| Scaridae | Chlorurus | spilurus | 180 |
| Scaridae | Hipposcarus | longiceps | 2 |
| Scaridae | Scarus | forsteni | 70 |
| Scaridae | Scarus | frenatus | 8 |
| Scaridae | Scarus | globiceps | 1 |
| Scaridae | Scarus | oviceps | 15 |
| Scaridae | Scarus | psittacus | 18 |
| Scaridae | Scarus | rubroviolaceus | 2 |
| Scaridae | Scarus | schlegeli | 3 |
| Scorpaenidae | Parascorpaena | picta | 3 |
| Sebastidae | Sebastiscus | marmoratus | 6 |
| Serranidae | Aethaloperca | rogaa | 6 |
| Serranidae | Cephalopholis | argus | 16 |
| Serranidae | Cephalopholis | boenak | 8 |
| Serranidae | Cephalopholis | leopardus | 4 |
| Serranidae | Cephalopholis | urodeta | 159 |
| Serranidae | Diploprion | bifasciatum | 1 |
| Serranidae | Epinephelus | fasciatus | 4 |
| Serranidae | Epinephelus | hexagonatus | 41 |
| Serranidae | Epinephelus | merra | 16 |
| Serranidae | Epinephelus | multinotatus | 10 |
| Serranidae | Epinephelus | quoyanus | 3 |
| Serranidae | Epinephelus | spilotoceps | 1 |
| Serranidae | Gracila | albomarginata | 3 |
| Siganidae | Siganus | fuscescens | 45 |
| Siganidae | Siganus | puellus | 1 |
| Siganidae | Siganus | vulpinus | 2 |
| Sparidae | Acanthopagrus | schlegelii | 1 |
| Tetraodontidae | Arothron | hispidus | 2 |
| Tetraodontidae | Canthigaster | valentini | 5 |
| Tetraodontidae | Takifugu | poecilonotus | 2 |
| Tetrarogidae | Paracentropogon | rubripinnis | 3 |
| Zanclidae | Zanclus | cornutus | 38 |

**S Table. 4** List of corals used for carbon content analysis

| **Family** | **Genus** | **Species** | **Sample Size** |
| --- | --- | --- | --- |
| Agariciidae | Pavona | danai | 1 |
| Agariciidae | Pavona | duerdeni | 3 |
| Agariciidae | Pavona | frondifera | 1 |
| Agariciidae | Pavona | maldivensis | 1 |
| Agariciidae | Pavona | varians | 9 |
| Merulinidae | Cyphastrea | agassizi | 1 |
| Merulinidae | Cyphastrea | microphthalma | 1 |
| Merulinidae | Cyphastrea | serailia | 1 |
| Merulinidae | Dipsastraea | amicorum | 1 |
| Merulinidae | Dipsastraea | matthaii | 1 |
| Merulinidae | Dipsastraea | truncata | 2 |
| Pocilloporidae | Seriatopora | hystrix | 4 |
| Poritidae | Porites | cylindrica | 4 |
| Poritidae | Porites | lutea | 6 |
| Poritidae | Porites | nigrescens | 1 |
| Poritidae | Porites | rus | 1 |
| Poritidae | Porites | tuberculosus | 2 |
